# Tumor heterogeneity in VHL drives metastasis in clear cell renal cell carcinoma

**DOI:** 10.1038/s41392-023-01362-2

**Published:** 2023-04-17

**Authors:** Junhui Hu, Ping Tan, Moe Ishihara, Nicholas A. Bayley, Shiruyeh Schokrpur, Jeremy G. Reynoso, Yangjun Zhang, Raymond J. Lim, Camelia Dumitras, Lu Yang, Steven M. Dubinett, Parmjit S. Jat, Jacques Van Snick, Jiaoti Huang, Arnold I. Chin, Robert M. Prins, Thomas G. Graeber, Hua Xu, Lily Wu

**Affiliations:** 1 Department of Molecular and Medical Pharmacology, David Geffen School of Medicine, University of California Los Angeles, Los Angeles, CA 90095 USA; 2 Department of Urology, West China Hospital, Chengdu, China; 3 Department of Hematology and Oncology, University of California San Diego, San Diego, CA 92103 USA; 4 Department of Neurosurgery, David Geffen School of Medicine, University of California Los Angeles, Los Angeles, CA 90095 USA; 5 Department of Biological Repositories, Department of Urology, Zhongnan Hospital of Wuhan University, Wuhan, China; 6 Department of Medicine, David Geffen School of Medicine, University of California Los Angeles, Los Angeles, CA 90095 USA; 7 Jonsson Comprehensive Cancer Center, David Geffen School of Medicine at UCLA, University of California Los Angeles, Los Angeles, CA 90095 USA; 8 MRC Prion Unit at UCL, Institute of Prion Diseases, 33 Cleveland Street, London, W1W 7FF UK; 9 Ludwig Institute for Cancer Research Ltd, Brussels, Belgium; 10 Department of Pathology, Duke University, Durham, NC USA; 11 Department of Urology, David Geffen School of Medicine, University of California Los Angeles, Los Angeles, CA 90095 USA; 12 Cancer Precision Diagnosis and Treatment and Translational Medicine Hubei Engineering Research Center, Wuhan, China

**Keywords:** Tumour heterogeneity, Cancer genetics

## Abstract

Loss of function of the von Hippel-Lindau (VHL) tumor suppressor gene is a hallmark of clear cell renal cell carcinoma (ccRCC). The importance of heterogeneity in the loss of this tumor suppressor has been under reported. To study the impact of intratumoral VHL heterogeneity observed in human ccRCC, we engineered *VHL* gene deletion in four RCC models, including a new primary tumor cell line derived from an aggressive metastatic case. The *VHL* gene-deleted (VHL-KO) cells underwent epithelial-to-mesenchymal transition (EMT) and exhibited increased motility but diminished proliferation and tumorigenicity compared to the parental VHL-expressing (VHL^+^) cells. Renal tumors with either VHL^+^ or VHL-KO cells alone exhibit minimal metastatic potential. Combined tumors displayed rampant lung metastases, highlighting a novel cooperative metastatic mechanism. The poorly proliferative VHL-KO cells stimulated the proliferation, EMT, and motility of neighboring VHL^+^ cells. Periostin (POSTN), a soluble protein overexpressed and secreted by VHL non-expressing (VHL^−^) cells, promoted metastasis by enhancing the motility of VHL-WT cells and facilitating tumor cell vascular escape. Genetic deletion or antibody blockade of POSTN dramatically suppressed lung metastases in our preclinical models. This work supports a new strategy to halt the progression of ccRCC by disrupting the critical metastatic crosstalk between heterogeneous cell populations within a tumor.

## Introduction

Renal cell carcinoma (RCC) ranks amongst the top ten most prevalent malignancies in the world. Annually diagnosed cases exceed 70,000 in the US and 350,000 worldwide.^1^ The clear cell subtype of RCC (ccRCC) constitutes more than 70% of RCC and features tumor cells with clear, lipid-laden cytoplasm. Patients with localized disease have a favorable 5-year survival of 73%. Unfortunately, over 30% of patients develop metastatic disease, frequently spreading to the lung.^2^ Despite treatment with new targeted and immune therapies, patients with metastatic ccRCC still have a poor outcomes with a median progression-free survival of 15.1 months.^3^

Detailed studies of von Hippel-Lindau (VHL) disease (a rare hereditary cancer syndrome manifesting as renal, CNS, adrenal, and pancreatic tumors) led to the cloning of the *VHL* tumor-suppressor gene.^4,5^ Somatic mutations of *VHL* were also identified in as many as 90% of sporadic, non-familial ccRCC cases.^6–10^ VHL protein functions as an E3 ubiquitin ligase that targets the alpha subunit of hypoxia-inducible transcription factors (HIF-αs) for oxygen-dependent degradation.^11,12^ Although the functional inactivation of VHL and constitutive activation of the HIF pathway have been implicated as the oncogenic driver,^13,14^ the precise oncogenic mechanism in ccRCC remains elusive. Numerous mouse models of renal-tubule-targeted *VHL* deletion failed to generate renal lesions beyond preneoplastic cysts.^15–19^ The loss of VHL function upregulates both HIF-1α and HIF-2α. However, these two paralogs have distinct, and often contrary, roles in their gene regulatory activities.^20,21^ A conundrum in the field is the opposing oncogenic roles of HIF-1α and HIF-2α in ccRCC.^22–24^

The contribution of VHL loss to metastasis is unknown, as there is no correlation between *VHL* status and clinical outcome.^25^ We and others have reported that silencing the *VHL* gene consistently results in epithelial-to-mesenchymal transition (EMT).^26–28^ EMT is an embryonic program reminiscent of the process that carcinomas adopt during metastatic spread,^29^ although the direct role of EMT in cancer metastasis is debated.^30,31^

Our *VHL*-deleted ccRCC models revealed a new concept: EMT contributes to metastatic dissemination, but indirectly. Specifically, the inactivation of VHL renders VHL^−^ tumor cells highly motile but non-proliferative. Yet, these VHL^−^ cells drive metastasis by stimulating the growth of VHL-expressing (VHL^+^) cells and producing soluble metastatic mediators, such as periostin (POSTN). POSTN enhances the motility of VHL^+^ cells and the dissemination of tumor cells by disrupting the vasculature. This cooperative mechanism of metastasis, occurring between two distinct cell populations, highlights the active role of intratumoral heterogeneity in cancer aggression. This study seeks to understand the metastatic crosstalk at play between the VHL^+^ and VHL^−^ tumor cells in ccRCC.

## Results

### Intratumoral heterogeneity in VHL expression is prevalent in ccRCC

**Fig. 1:**
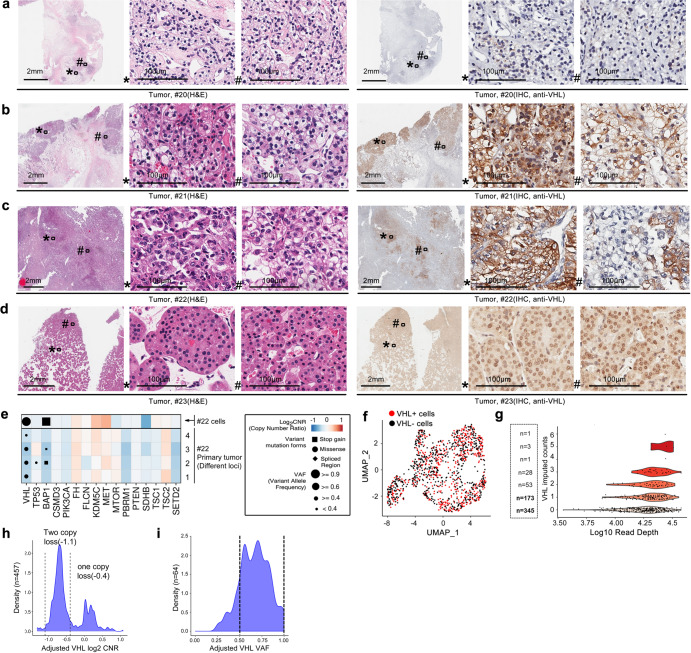
Human ccRCC tumor specimen showed intratumoral heterogeneity in VHL expression. H&E stain and VHL IHC performed on parallel sections of tumor from different cases of human RCC are shown in (**a**) case #20, (**b**) case #21, (**c**) case #22 and (**d**) case #23. Scale bar for low magnification field: 2 mm; for high magnification field: 100 μm. **e** Mutational data from WES of common oncogenic driver genes in ccRCC are shown for four loci of the patient’s tumor and the derived cell line of case #22. Point sizes represent variant allele frequencies. Values above 0.4 represent likely clonal mutations and above 0.9 represent clonal mutations combined with loss of heterozygosity. Colors represent log2 CNRs for each gene, with DNA gains in red and losses in blue. **f** UMAP dimensionality reduction plot of single-cell sequencing from the case #22 primary tumor tissue and disassociated primary tumor cells colored by VHL expression positivity in each cell. A total of 1343 P0 (passage 0) #22 tumor cells were analyzed. **g** Scatterplot of sequencing depth vs. VHL imputed gene expression across non-cycling single cells from single cell sequencing in the case #22 with the number of cells at each expression level of VHL listed. **h** Density plot showing the CNRs of the VHL locus in the TCGA-KIRC cohort (*n* = 459) after adjusting for both tumor purity and ploidy with consensus estimates of purity. A CNR value of −1.1 represents a two-copy loss of VHL (dotted line) and −0.4 indicates one copy loss. **i** Density plot showing the VAF of somatic VHL mutations in the TCGA-KIRC cohort (*n* = 148) after adjusting for both tumor purity and ploidy (with consensus estimates of tumor purity)

The VHL gene mutation is recognized as an early oncogenic event in ccRCC.^9^ However, it is unclear whether the complete loss of VHL function is uniform throughout the entire tumor. VHL protein expression was analyzed by immunohistochemistry (IHC) of large tissue sections of 25 ccRCC cases and one renal oncocytoma (Table 1 and Supplementary Fig. 1a). The most recent 10 cases (#17 to 26) were collected from consecutive surgeries performed by a single surgeon (Table 1 and Supplementary Fig. 1b). Intratumoral heterogeneity in VHL expression was common as none of the cases displayed a uniform loss of VHL (Table 1 and Supplementary Fig. 1a). Representative images of H&E and VHL stains are shown in Fig. 1a–d. Case #20 contained the least amount of VHL positivity of 10%, while case #23 is a benign oncocytoma that is uniformly VHL-positive.

**Table 1:**
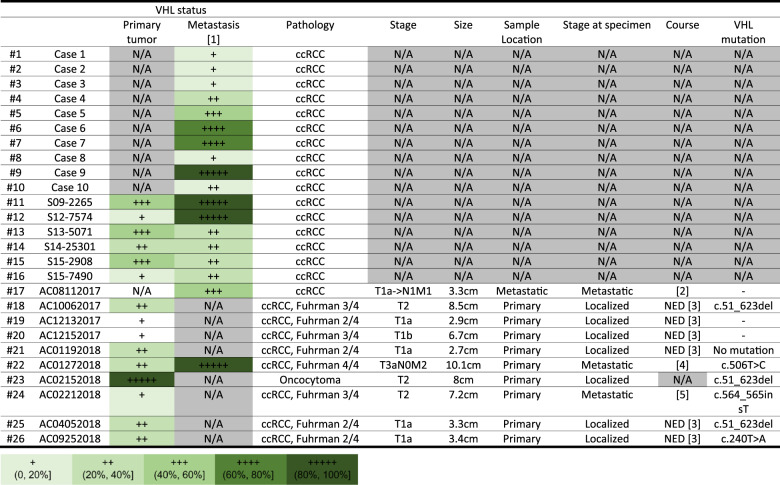
VHL status summary of human ccRCC samples

[1]: Metastasis as mentioned in this form includes both local invasion and lymph node metastasis

[2]: 65 y/o, female, the sample is a recurrent retroperitoneal lymph node metastasis resected 3 months after the patient received partial nephrectomy for her left renal mass

[3]: No evidence disease

[4]: 59 y/o, male, the sample is a primary renal mass from the right kidney in the first surgery. The patient presented with 10 cm right renal mass and bilateral pulmonary nodules that were biopsy proven lung metastases. He underwent cytoreductive nephrectomy in the first surgery and received 5 months of cabozantinib until progression, then axitinib and ipilimumab/nivolumab for another 4 cycles and nivolumab maintenance therapy until death 1 year after the first surgery.

[5]: The patient is stable on cabozantinib

To investigate *VHL* and other oncogenic mutations of ccRCC, we established primary tumor cell lines and patient-derived xenografts (PDXs) in the chorioallantoic membrane (CAM) model from surgical specimens (cases #18-26). The rate of PDX engraftment in CAM is 80%.^32^ Here, we focused on the most aggressive metastatic ccRCC, case #22 (Table 1). This patient presented with a 10-cm, Fuhrman grade 4, primary tumor and bilateral lung metastases and succumbed within one year of nephrectomy despite multiple interventions. As shown in Fig. 1c, VHL expression in the primary tumor of #22 was highly heterogeneous, with VHL^+^ cells juxtaposed to VHL^−^ cells. The CAM PDXs established from small tumor pieces of the primary tumor from case #22 revealed the presence of intermixed VHL^+^ and VHL^−^ tumor cells (Supplementary Fig. 1c). A primary tumor cell line was generated from case #22, which consisted of an equal mixture of VHL^+^ and VHL^−^ tumor cells upon initial tumor dissociation (P0). However, only the VHL^+^ tumor cells were able to propagate continually, up to passage 20 currently (data not shown). Whole exome sequencing (WES) showed that the #22 cell line shared more than 80% of COSMIC annotated mutations found in the parental tumor (Supplementary Fig. 1d). The *VHL* sequence of the #22 cell line possessed a homozygous in-frame T506C transition, leading to an L169P amino acid substitution (Supplementary Fig. 1e), which is a common variant^33^ that altered a surface amino acid near the C-terminus. We showed this L169P variant is comparable to wildtype VHL in its protein stability and in its ability to degrade HIF-1α protein (Supplementary Fig. 1f–i) and its hypoxia regulatory function assessed by three hypoxia signatures (Supplementary Fig. 1j). Our functional analyses reach the same conclusion as an earlier report by Rechsteiner et al.^34^ that VHL L169P is a passenger mutation which does not cause ccRCC.

To explore the contribution of common ccRCC driver mutations (such as *VHL*, *TP53*, *BAP1*, *PBRM1*, and *SETD2*) in case #22, four different areas of the primary tumor were analyzed by WES and compared to its derivative cell line (Fig. 1e). Variant allele frequencies (VAFs) and copy number ratios (CNR) showed clonal missense mutations in the *VHL* gene and frameshift mutations in the *BAP1* gene in the #22 cell line. However, these two mutations were subclonal, with varying contributions in the four tumor areas, supporting cellular heterogeneity within the primary tumor.

Next, we analyzed single-cell sequencing of primary tumor tissue and dissociated primary tumor cells from case #22. Heterogeneous VHL positivity was confirmed (Fig. 1f, g), which cross-validates the VHL staining in Fig. 1c demonstrating the presence of both VHL-positive and VHL-negative populations in the tumor. Next, we utilized the TCGA database to further investigate VHL heterogeneity. The CNR at the *VHL* locus was analyzed in the TCGA KIRC (Kidney Renal Clear Cell Carcinoma) cohort (*n* = 459) after adjusting for both tumor purity and ploidy. The peak CNR value (average for the mixture of cells analyzed) was between −1.1 and −0.4, suggesting that the purity and ploidy of corrected TCGA samples had a subclonal, single-copy loss of *VHL*, as a value of −1.1 represents a two-copy loss and −0.4 represents a one-copy loss (shown as the dotted lines, Fig. 1h). The VAFs of somatic *VHL* mutations in the TCGA KIRC cohort (*n* = 148), after adjusting for tumor purity and ploidy, also displayed a wide spectrum spanning between 0.5 and 1, indicating subclonal mutations (Fig. 1i). These results use consensus purity and ploidy estimates from three computational algorithms and IHC analysis (see Supplementary Methods). Further evidence of subclonal VHL copy number loss can be found by adjusting with only one of the computational measures of tumor purity (ABSOLUTE) (Supplementary Fig. 1k, l).

Collectively, IHC and genomic profiling indicates that VHL protein expression and gene mutation is heterogenous within individual human ccRCC tumors.

### Metastasis requires cooperation between RCC cells with and without wild-type VHL expression

**Fig. 2:**
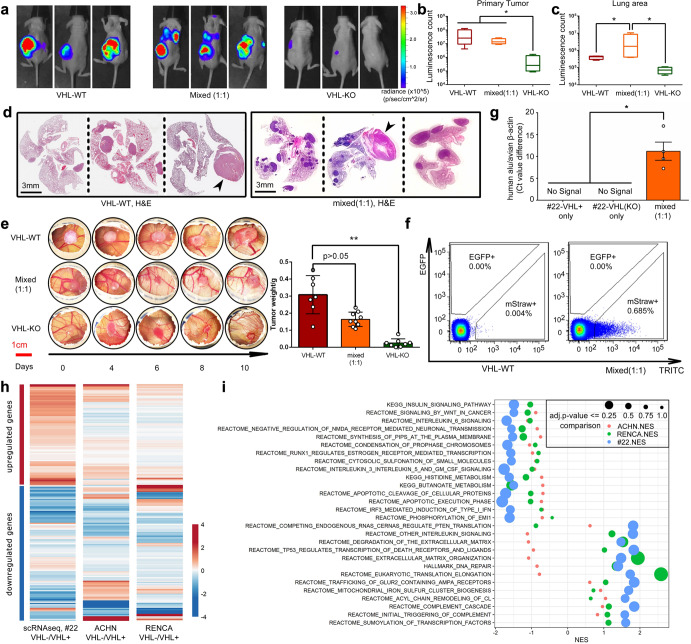
VHL-KO cells cooperate with VHL-WT cells to cause metastasis. **a** Mice were implanted with a total of 1 × 10^6 tumor cells, either RC-VHL-WT, RC-VHL-KO, or a 1:1 mixture of the two cell types, into their left kidney (*n* = 6 per group). Images of three representative animals assessed by BLI on week 4 post-implantation are shown. **b** Primary tumor indicated by BLI in different groups are shown at the end point. **c** Lung metastases are indicated by elevated BLI signals in the thoracic cavity in the mixed tumor group. One-way ANOVA was used to test the statistical significance in (**b**) and (**c**) with *n* = 6, presented with mean ± SD. **d** H&E-stained sections (low magnification) of lung and heart of each animal in the RC-VHL-WT tumor group and mixed (1:1) tumor group. Arrowhead indicates the heart. Scale bar: 3 mm. **e** RC tumor growth was assessed in the CAM tumor system through longitudinal observation. Day 0 is the day of tumor cell implantation, which occurred on day 7 postfertilization. Scale bar: 1 cm. One-way ANOVA was used in the comparison with *n* = 9, presented with mean ± SD. **f** Flow cytometric analyses of CTC from chick embryos, bearing either VHL-WT cells (marked with mStrawberry) or 1:1 VHL-WT and VHL-KO (marked with EGFP) mixed CAM tumor, harvested on day 21 post-fertilization. **g** In the CAM tumor system, the metastasis in the duck liver can only be seen in mixed implantation of primary tumor cells from #22 and its VHL-KO counterpart as analyzed by TaqMan probes for human alu sequence and avian β-actin. *n* = 5 with “no signal” sample dots not shown and Ct value differences are presented with mean ± SEM. **h** Heatmap of the top 200 genes based on conserved gene expression patterns across comparisons of #22 VHL^+^ vs. VHL^−^ cells in single-cell sequencing (as noted in Fig. 1f), VHL KO vs. VHL WT ACHN models, and VHL KO vs. VHL WT RENCA models. Heatmap colors represent log2 fold changes *z*-score scaled by column. Detailed information is listed in Supplementary Table 2. **i** Top 30 gene sets based on conserved enrichment scores across the same three comparisons are listed. The color of points represents the differential expression comparison (ACHN VHL-KO vs VHL-WT as red, RENCA VHL-KO vs VHL-WT as green, and the single cell comparison of #22-VHL^+^ vs VHL^−^ as blue) and the size of the points corresponds to the adjusted *p*-value from GSEA, in which the bigger circles indicate up/down-regulation with lower *p*-values. (*p < 0.05, **p < 0.01) (not consistent with adjusted *p* vals in Fig. 2i)

To study the functional interaction between VHL^+^ and VHL^−^ RCC cells, we deleted the *VHL* gene by CRISPR/Cas9 in one murine and three human RCC models, including the primary cell line #22 reported here. The first clonal *VHL*-deleted murine RENCA line is denoted as RC-VHL-KO, and the parental, VHL^+^ control treated line is denoted as RC-VHL-WT. Renal tumors were established in mice with either RC-VHL-WT cells, RC-VHL-KO cells, or a 1:1 mixture of the two cell lines. The growth and dissemination of these tumors were monitored by bioluminescence imaging (BLI) using firefly luciferase. RC-VHL-WT and mixed primary tumors grew well, but VHL-KO tumors grew poorly (Fig. 2a, b). Only the mixed tumor bearing mice exhibited prominent thoracic metastasis (Fig. 2a, c), and they suffered tumor cachexia (Supplementary Fig. 2a). Lung metastasis was not observed in the VHL-KO group (Supplementary Fig. 2b). Histological analyses revealed that the mixed-tumor group exhibited greatly increased numbers and sizes of lung metastases compared to the VHL-WT group (Fig. 2d).^35^ The in vivo growth and metastatic behavior of the RC tumors were further verified in the CAM tumor system,^36,37^ substantiating the poor growth of VHL-KO tumors (Fig. 2e) and the heightened metastatic spread of the mixed tumors with increased circulating tumor cells (Fig. 2f).

Next, we ascertained whether this novel cooperative mechanism of metastasis could be operating in human tumor models. The same CRISPR/Cas9 lentiviral system was employed to knock out the *VHL* gene in the human cell line ACHN (AC),^28^ Caki-1^38^ and the primary tumor cell line of case #22. The AC model is a widely used human RCC line, expressing wild-type VHL protein.^39^ However, it was recently reclassified as a papillary RCC and not ccRCC.^38^ The AC-VHL-KO line exhibited an EMT cellular morphology, elevated expression of EMT markers, and slowed growth (Supplementary Fig. 2c, d). Consistent with the RC model, renal tumors with AC-VHL-WT or 1:1 mixed of VHL-WT and VHL-KO cells grew well, but lung metastasis was observed only in mice bearing the mixed-tumor group (Supplementary Fig. 2e). Caki-1 is a ccRCC cell line expressing wildtype VHL protein.^38^ Analogous to findings in RC and AC, the deletion of *VHL* gene in Caki-1 resulted in upregulation of EMT markers, including POSTN^28^ (Supplementary Fig. 2f).

The CRISPR/Cas9-mediated biallelic deletion of VHL gene in the VHL^+^ primary tumor cells of case #22, designated as #22 VHL-KO cells, was confirmed by DNA sequencing (data not shown). CAM tumors were established for #22 VHL^+^ primary cells, #22 VHL-KO or a 1:1 mixture of these two cell types (Supplementary Fig. 2g). Importantly, the metastatic potential of the #22 CAM tumors, assessed by the presence of tumor cells in the duck embryo, was only seen in the mixed tumor group (Fig. 2g). Further analyses of differential single-cell gene expression in VHL-KO/VHL^+^ cells for these three models, #22, ACHN and RENCA, showed a congruent 200-geneset pattern of up- and down-regulation for a wide spectrum of genes (Fig. 2h). Congruent patterns of functional enrichment, such as sumoylation of transcription factors, and depletion, such as wnt signaling pathway and apoptotic cleavage of cellular proteins, were observed across these three RCC models (Fig. 2i).

Collectively, the data from the *VHL*-deleted RCC models, #22, ACHN and RENCA, revealed that the cooperative interactions between two distinct populations of tumor cells (VHL^−^ and VHL^+^ cells) are required to produce distant metastases.

### VHL^−^ cells induce the proliferation of VHL^+^ tumor cells

**Fig. 3:**
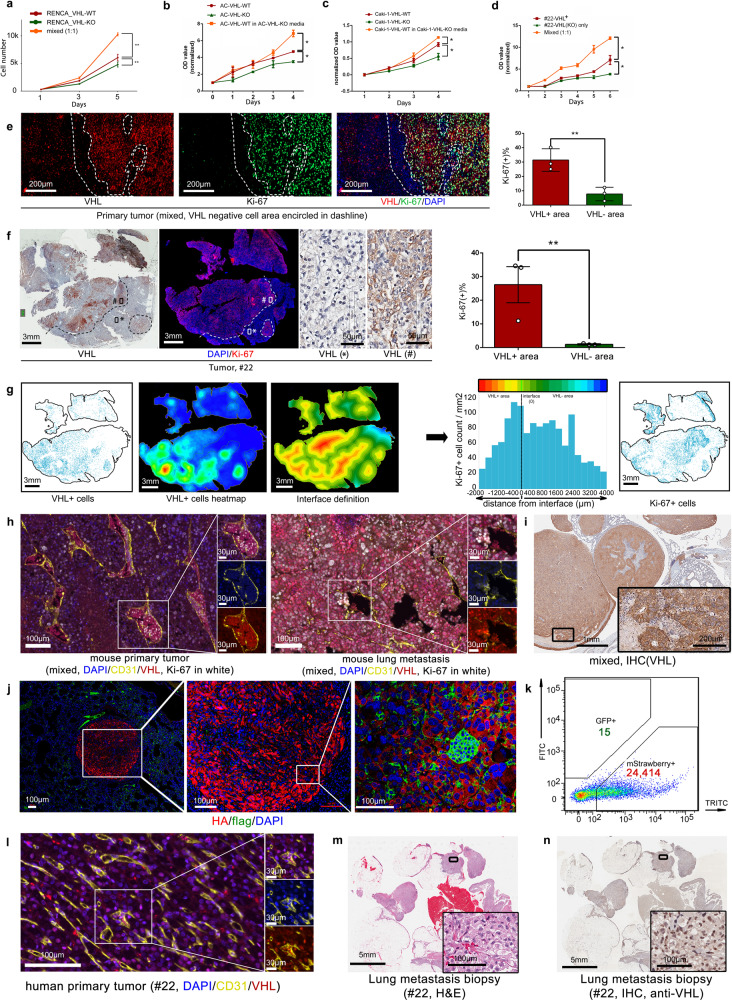
VHL^−^ cells induce the proliferation and the metastasis of VHL^+^ cells. **a** The growth rate of RC-VHL-WT (red line), RC-VHL-KO cells (green line), and a 1:1 mixture of the two cells (orange line) in a transwell setting. **b** The growth rate of ACHN VHL-WT cells (red line), its VHL-KO counterpart (green line) or VHL-WT cells with the addition of VHL-KO condition media (orange line). **c** The growth rate of the Caki-1 -VHL-WT (red line), Caki-1-VHL-KO (green line), and the Caki-1-VHL-WT in the conditioned media from Caki-1-VHL-KO (orange line). **d** The growth rate of the VHL + primary cell line from case #22 (red line), its VHL-KO derivative (green line) and VHL + co-culture with its VHL-KO derivative in a transwell setting. One-way ANOVA was used in the comparisons in (**a**), (**b**), (**c**) and (**d**) with triplicate repeats, presented with mean ± SD. **e** A section of primary tumor derived from implantation of 1:1 RC-VHL-KO:RC-VHL-WT cells was stained with IF to detect VHL (red), Ki67 (green), and nuclei (DAPI, blue). The dash lines demarcate VHL-negative areas with intact nuclei. Scale bar: 200 μm. **f** VHL IHC and Ki-67 IF in serial sections of human ccRCC (case #22). High-magnification images show cytoplasmic VHL expression specifically in area (#) and not in area (*). The bar graph shows the average percentage of Ki-67 positivity in the VHL-positive and VHL-negative regions. Scale bar for low magnification field: 3 mm; for high magnification field: 50 μm. Student *t*-test was used in the comparisons (**e**) and (**f**) with triplicate repeats and presented with mean ± SD. **g** The spatial relationship between VHL and Ki-67 expression in case #22 was assessed with IF. Fluorescent images were analyzed with HALO software. In the first and fifth panels, VHL-positive cells and Ki-67-positive cells are represented by blue dots, respectively. The second panel shows a heatmap of VHL-positive cell density. The third panel shows a boundary map of the VHL-positive tumor regions as topographic contour lines indicating the distance from the tumor boundary. For distance measurements of Ki67-positive cells, contour lines were placed up to 2000 μm from the tumor edge toward the inside of the tumor and up to 4000 μm away from the tumor edge of VHL-positive tumor regions. Regions between the contour lines are shown as different colors from the innermost red to farthest blue. Ki-67-positive cells in each region were counted, normalized to the area, and plotted in a histogram that is shown in the fourth panel. Scale bar: 3 mm. **h** IF staining of the CD31 (yellow), VHL (red), Ki-67(white) as well as DAPI (blue) in mixed implanted RENCA-VHL-WT and VHL-KO cells mouse tissues. Left: the primary tumor. Right: lung metastasis. Scale bar for the low magnification field: 100 μm; for high magnification field: 30 μm. **i** VHL IHC staining of a large, lung metastasis from the mixed implanted group with RC-VHL-WT and RC-VHL-KO cells. Scale bar for low magnification field: 1 mm; for high magnification field: 200 μm. **j** IF of a small, lung metastatic nodule from a mouse implanted with the mixture with HA-positive RC-VHL-WT cells shown in red and the few flag-positive VHL-KO cells shown in green. Scale bar: 100 μm. **k** Flow cytometry analysis of the lung metastasis showing the relative proportion of RC-VHL-WT (TRITC+) and RC-VHL-KO(FITC+) cells. **l** IF of CD31(yellow), VHL (red) and DAPI (blue) on the primary tumor tissue from the case #22. Scale bar for low magnification field: 100 μm; for high magnification field: 30 μm. **m** H&E staining and (**n**) VHL IHC staining of the lung metastasis from the case #22. Scale bar for the low magnification field: 5 mm; for high magnification field: 100 μm. (*p < 0.05, **p < 0.01)

An immediate question raised by the cooperative metastatic model (Fig. 2) is the nature of the crosstalk between the two cell populations that is inducing the metastasis. In the murine RC,^35^ the human AC,^28^ Caki-1, and #22 primary tumor cell models, the derivative VHL-KO line of all four models grew slower than their parental VHL^+^ cells in cell culture (Fig. 3a–d). Remarkably, all four models exhibited a similar growth induction of VHL^+^ parental cells in the presence of VHL-KO cells in transwell co-cultures (Fig. 3a, d) or with exposure to conditioned media (Fig. 3b, c). These results implicate the influence of soluble factors from VHL-KO cells in promoting VHL^+^ proliferation.

Following the in vitro findings presented above, we evaluated the in vivo impact of this crosstalk. Cellular proliferation, assessed by the rate of Ki67 staining, was more than three times higher in the VHL-positive (31.4%) than the VHL-negative areas (7.8%) of the mixed RC renal tumor (Fig. 3e) and lung metastases (Supplementary Fig. 3a, b). Analysis of human ccRCC tumor #22 revealed the same pattern, with the Ki67 positivity rate more than seven times higher in the VHL-positive (26.6%) than the VHL-negative tumor areas (1.4%) (Fig. 3f). The spatial relationship assessed by HALO infiltration analysis revealed that the highest concentration of Ki-67^+^ cells resided at the edges of VHL^+^ areas were in the closest proximity to the VHL^−^ cells (Fig. 3g). This finding supports the in vitro findings that VHL^−^ cells exert paracrine influence to promote the proliferation of VHL^+^ cells.

We next queried the involvement of the VHL^+^ cells in the metastatic process. Longitudinal analysis of circulatory tumor cells (CTCs) in mice bearing mixed RC renal tumors revealed that VHL-WT was the predominant population of tumor cells that escaped into the circulation, especially at later times after 4 weeks of tumor growth (Supplementary Fig. 3c). Congruent with the CTC results, multiplex immunofluorescence (MIF) stains revealed that intravascular tumor cells in the RC primary tumors and lung metastases were predominantly VHL^+^ cells (Fig. 3h and Table 2). Furthermore, the majority of the intravascular VHL^+^ cells were proliferative (Ki67^+^, Table 3), including a pocket of extravasating tumor cells located in the perivascular area (Fig. 3h). These intravascular Ki67^+^, VHL^+^ cells likely continued to grow and expand to form the rampant metastases in the lungs, since the metastatic lesions were heavily dominated by proliferating VHL^+^ cells (Supplementary Fig. 3a, b). The high prevalence of VHL^+^ cells was observed in large (Fig. 3i) and very small lung metastatic lesions (Fig. 3j), and the proportion of VHL^+^ cells in metastases exceeded 99% (Fig. 3k). Paralleling the preclinical scenario, the MIF stain of the #22 primary tumor showed a predominance of VHL^+^ cells within the vasculature (Fig. 3l). IHC of the lung metastases of case #22 showed a high prevalence of VHL^+^ cells, far exceeding the 40% VHL positivity in the primary tumor (Fig. 3m, n and Table 1).

**Table 2:** VHL positivity in intravascular tumor cells (RENCA mixed tumor and human primary tumor, #22)

	mouse		human
	primary tumor	lung metastasis	primary tumor
intravascular VHL + cells	1742	349	278
intravascular VHL- cells	273	94	86

**Table 3:** Ki67 positivity in intravascular VHL + tumor cells (RENCA mixed tumor and human primary tumor, #22)

mouse primary tumor (RENCA)		human primary tumor (#22)	
intravascular VHL + cells		intravascular VHL + cells	
Ki67+ cells	Ki67- cells	Ki67+ cells	Ki67- cells
1366	376	220	58

Collectively, our preclinical and clinical findings suggest that VHL-KO or VHL^−^ tumor cells drive cooperative metastasis by inducing the proliferation of VHL-WT or VHL^+^ cells.

### VHL^−^ cell production of HIF-1α target periostin drives EMT and motility of VHL^+^ tumor cells

Four HIF-1α-regulated genes (*POSTN*, *TNFSF13B*, *PPEF1* and *SAMSN1*) were identified to be over-expressed upon VHL gene deletion in RC-VHL-KO cells in our previous study.^28^ These four genes could likely contribute to metastatic progression since their coordinate upregulation in patients’ tumor predicted a very poor survival in the TCGA RCC (KIRC) database.^28^ Amongst these four genes, we focused on *POSTN* because it encodes a secreted cell-adhesion protein upregulated in EMT that correlates with aggressive cancers,^40,41^ including RCC^42^ (Supplementary Fig. 4a). However, POSTN’s oncogenic role in RCC is undefined.

**Fig. 4:**
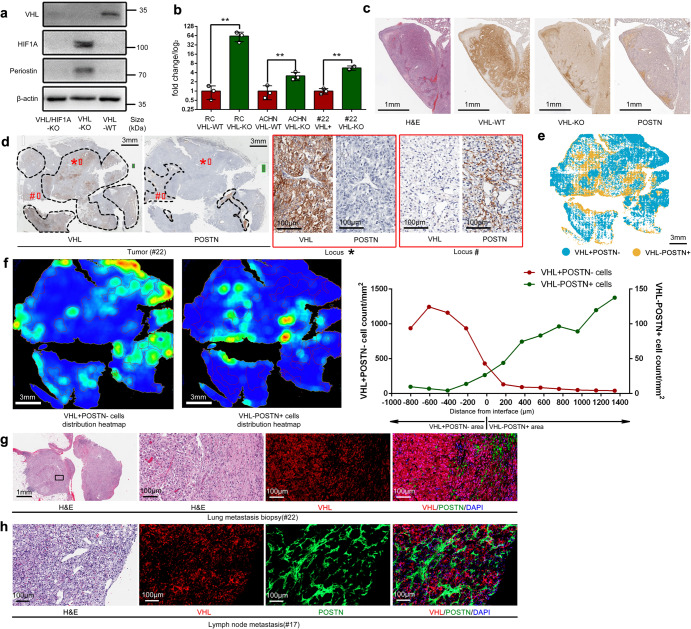
POSTN is overexpressed in VHL-KO or VHL^−^ cells in tumors. **a** Western blot assessing the expression of VHL, HIF1A and POSTN protein in RENCA VHL-WT, VHL-KO and VHL/HIF1A double knockout cells. **b** qRT-PCR of POSTN RNA in RENCA, ACHN and the patient-derived primary cells from case #22 and the VHL-deleted derivative (VHL-KO) of each. Student t-test was used in the comparison with triplicate repeats and presented with mean ± SD. **c** Serial sections from a large, lung metastatic lesion resulting from implantation of a 1:1 mixture of VHL-WT and VHL-KO cells were stained with H&E and IHC against HA tag (VHL-WT cells), flag tag (VHL-KO cells), and anti-POSTN. Scale bar: 1 mm. **d** Serial sections of the primary tumor tissue from case #22 were stained for VHL and POSTN. Dotted encircled areas are VHL-positive cell aggregated areas (left) and POSTN positive aggregated areas (right). Higher magnification of the boxed areas (**a**) and (**b**) are shown on the right. Scale bar for low magnification field: 3 mm; for high magnification field: 100 μm. **e** Multiplex IF stain, analyzed by HALO software, showing the cellular distribution of VHL^+^ POSTN^−^ and VHL^−^POSTN^+^ cells in case #22 primary tumor. Scale bar: 3 mm. **f** HALO analysis in (**e**) was used to generate heatmap of VHL^+^ POSTN^−^ and VHL^−^POSTN^+^ cells. Warmer colors (orange) identify areas of denser cells, and cooler colors (dark blue) signify areas with sparser cells. The rightmost plot scored the VHL^+^ POSTN^−^ and VHL^−^POSTN^+^ cells in each evenly divided area with respect to the interface border of the VHL^+^ POSTN^−^ area. It shows VHL^+^ POSTN^−^ cells (red curve, left *y*-axis) and VHL^−^POSTN^+^ cells (green curve, right *y*-axis) do not co-localize in the same area. Scale bar: 3 mm. **g** The lung metastatic lesion of case #22 and (**h**) the retroperitoneal lymph node metastatic lesion of case #17 were stained by H&E and IF to detect VHL (red), POSTN (green), and nuclei, (DAPI, blue). Scale bar for the low magnification field: 1 mm; for the high magnification field: 100 μm. (*p < 0.05, **p < 0.01)

We determined that POSTN is a direct transcriptional target of HIF1α but not HIF2α (Supplementary Fig. 4b–e and Fig. 4a). The expression of POSTN was upregulated upon VHL deletion and HIF1α upregulation in RENCA, ACHN, and the #22 line at the protein (Fig. 4a) and RNA levels (Fig. 4b). Importantly, the upregulation of POSTN in VHL-KO or VHL^−^ cells was observed in vivo in mouse or human tumors and metastatic tissues. As shown in Fig. 4c, POSTN colocalized with RC-VHL-KO cells but not with RC-VHL-WT cells, in a lung metastatic lesion. IHC of VHL and POSTN in serial sections of the primary tumor of case #22 (Fig. 4d) showed that VHL^+^ areas were POSTN negative (POSTN^−^) whereas VHL^−^ areas stained positive for POSTN (POSTN^+^) (Fig. 4e). Quantitative analysis of VHL^+^POSTN^−^ and VHL^−^POSTN^+^ cells in case #22 using the HALO image analysis software confirmed the distinct spatial distribution of these two populations (Fig. 4f). IF stain of the lung metastatic lesions of case #22 showed the predominance of VHL^+^ cells, which were excluded from the POSTN^+^ cells (Fig. 4g). This reciprocal relationship was further confirmed in the retroperitoneal lymph node metastasis of case #17 (Fig. 4h and Table 1). IHC analyses of a tissue microarray (TMA) constructed from over 300 tumor tissues from ccRCC patients^43^ also corroborated the reciprocal expression of VHL and POSTN (Supplementary Fig. 4f).

**Fig. 5:**
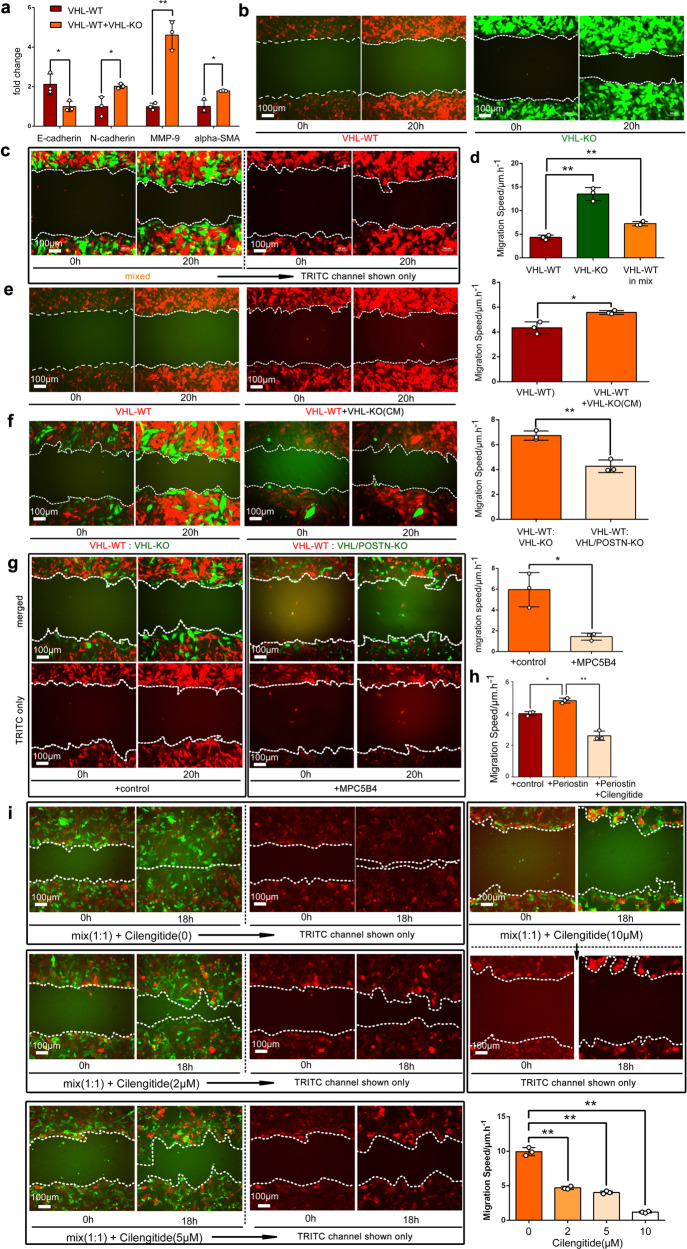
VHL-KO cells induce the EMT and motility of VHL-WT cells by POSTN. **a** EMT markers (E-cadherin, N-cadherin, MMP-9 and alpha-SMA) assessed by qRT-PCR in RC-VHL-WT cells alone or co-cultured with VHL-KO cells, separated by a transwell plate (VHL-WT + VHL-KO). **b**–**g** The motility of fluorescently marked RC cells was measured in a 2D scratch assay by time lapse live-cell microscopy, monitored over 20 h. **b** The motility of mStrawberry marked RC-VHL-WT cells and EGFP marked RC-VHL-KO cells were measured. **c** The motility of a 1:1 mixed culture of RC-VHL-WT and RC-VHL-KO cells (top panels). TRITC channel revealed the motility of RC-VHL-WT cells in the mixed culture (lower panel). Respective videos are in Supplementary Movie S1. **d** The quantified migration speed of the three cell groups is shown. **e** The migration speed of VHL-WT cells with (right) or without (left) the addition of conditioned medium of VHL-KO cells, cultured for 2 days at 90% confluence. Respective videos are shown in Supplementary Movie S3. **f** The migration speed of VHL-WT cells (mStrawberry^+^) co-cultured with VHL/POSTN-KO cells (EGFP^+^) or VHL-KO cells (EGFP^+^) was shown (also see Supplementary Movie S5). **g** Anti-POSTN mAb MPC5B4 (1 µg/mL) was added to VHL-WT cells co-cultured with VHL-KO cells. The migration speed of VHL-WT cells without and with MPC5B4 was shown (see Supplementary Movie S6). **h** The migration speed of VHL-WT cells alone or with the addition of recombinant POSTN protein or with the addition of POSTN and the integrin inhibitor cilengitide was assessed. **i** Scratch assay of a 1:1 mixture of VHL-WT and VHL-KO cells was assessed at 18 h. The migration speed of VHL-WT cells in the mixture was measured with 0, 2, 5 or 10 μM cilengitide added (see Supplementary Movie S7). Scale bar: 100 μm. Student *t*-test was used in (**a**), (**e**), (**f**) and (**g**) and one-way ANOVA was used in (**d**), (**h**) and (**i**) with triplicate repeats and presented with mean ± SD. (*p < 0.05, **p < 0.01)

Since POSTN has been implicated in EMT and metastasis,^39,40^ we surmised it could be a soluble mediator from VHL-KO cells that induces the EMT and motility of VHL-WT cells. Co-culture of RC-VHL-KO cells and RC-VHL-WT cells upregulated EMT markers such as *N-Cad*, *MMP-9*, and *SMA* and suppressed the epithelial marker *E-Cad* (Fig. 5a). Cellular motility was measured by time-lapse live-cell microscopy of mStrawberry-labeled RC-VHL-WT or EGFP-labeled RC-VHL-KO cells alone (Fig. 5b) or in co-cultures (Fig. 5c). VHL-KO cells migrated much faster than VHL-WT cells (Fig. 5d and Supplementary Movie S1a–d), and the migration of VHL-WT cells was greatly enhanced in co-culture with VHL-KO cells (Fig. 5d). A 3D migration assay through an extracellular matrix reaffirmed the results of the 2D assay (Supplementary Fig. 4g and Supplementary Movie S2a–d). Conditioned medium from VHL-KO cells also enhanced the motility of VHL-WT cells (Fig. 5e and Supplementary Movie S3), supporting soluble factor(s) produced by VHL-KO cells promoting cellular motility.

Two approaches were used to verify POSTN’s role in the paracrine crosstalk. First, we generated two double-gene knockout lines to abrogate POSTN expression: *VHL* and *HIF1α* (RC-VHL/HIF1A-KO, Fig. 4a) or *VHL* and *POSTN* (RC-VHL/POSTN-KO, Fig. 5f). Both of these double knockout lines exhibited significantly decreased augmentation of VHL-WT cells’ motility compared to VHL-KO cells (Supplementary Fig. 4h and Supplementary Movies S4a, b, S5a, b). Secondly, we employed a highly specific anti-POSTN monoclonal antibody (MPC5B4) to disrupt its functional interaction with integrin αVβ3.^44^ The POSTN blockade abrogated VHL-KO cells’ stimulation of VHL-WT cells’ motility (Fig. 5g and Supplementary Movie S6a, b). The addition of recombinant POSTN to VHL-WT cells significantly promoted their motility, which was blocked by the cyclic-peptide integrin inhibitor, cilengitide (Supplementary Fig. 4i and Fig. 5h). The addition of recombinant POSTN to VHL-WT cells activated focal adhesion kinase (FAK) via phosphorylation at Tyr397 but not Tyr925, which was blocked by cilengitide (Supplementary Fig. 4j). Cilengitide also disrupted the VHL-KO cells’ enhancement of VHL-WT cells’ motility in a dose-dependent manner (Fig. 5i and Supplementary Movie S7a–d). Taken together, these findings support VHL^−^ cells secreting POSTN to mobilize VHL-WT cells.

### Periostin enhances vascular destruction and tumor cell intravasation critical for metastasis

**Fig. 6:**
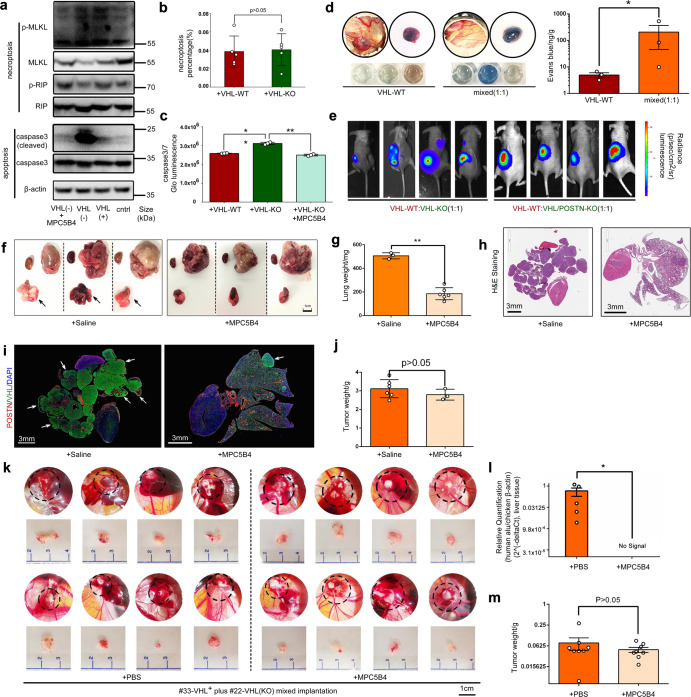
Blocking POSTN’s pro-metastatic activities, including vascular destruction, inhibited lung metastases. **a** HUVECs were co-cultured in a transwell setting with RC-VHL-WT (labeled “VHL (+)” here) or RC-VHL-KO (labeled “VHL(−)” here) cells without or with the anti-POSTN mAb MPC5B4. HUVECs were harvested after 48 h of culturing, and cell extracts were analyzed by western blot for necroptosis- and apoptosis-associated proteins. **b** HUVECs co-cultured with VHL-WT or VHL-KO cells for 48 h were assessed for necroptosis with a reporter assay by scoring the count of EthD-III(+) cells, which was normalized to the Hoechst 33342-positive nuclei count. **c** Apoptosis was evaluated with the Caspase-Glo 3/7 luminescence reporter assay in HUVECs co-cultured with VHL-WT cells, VHL-KO cells, or VHL-KO cells plus 1 µg/mL of anti-POSTN MPC5B4 mAb. **d** Tumor vascular leakage was assessed with the Miles assay on CAM tumors from VHL-WT cells or a 1:1 mixture of VHL-WT and VHL-KO cells. Evans Blue dye was injected intravenously into the chick embryo. The extent of tumor vascular leakage was scored by the amount of dye that leaked into the tumor. **e** Mice received intrarenal implantation of 1 × 10^6 total cells consisted of 1:1 mixture of RC-VHL-WT cells and RC-VHL-KO cells, or a 1:1 mixture of RC-VHL-WT and RC-VHL/POSTN-KO cells. BLI at 4 weeks post-implantation is shown. **f** Mice that received renal implantation of a 1:1 mixture of RC-VHL-WT and RC-VHL-KO cells were treated with either control IgG or anti-POSTN MPC5B4 mAb (*n* = 6). Scale bar: 1 cm. **g** Primary tumors and lungs harvested at 4 weeks post-implantation are shown with lung weights. **h** H&E stain of lung lobes and heart from control- or MPC5B4-treated tumor-bearing animals. Scale bar: 3 mm. **i** IF stain for POSTN in red, VHL in green, and DAPI in blue of the same tumor sections in (**h**). White arrows indicate selected lung metastases. Scale bar: 3 mm. **j** primary tumor weights for each group are shown. **k** Gross view of the primary tumor on CAM treated with either control or MPC5B4, and tumors were established from mixed implantation of the primary cell line from case #22 and its VHL-KO counterpart. Scale bar: 1 cm. The qRT-PCR relative quantification of the chicken liver metastasis is shown in (**l**), and the primary tumor weight analysis is shown in (**m**) as mean ± SEM. Student *t*-test was used in (**b**) and (**d**) with triplicate repeats, in (**g**), (**j**) and (**l**) with *n* = 6 (“no signal” sample dots not shown, Ct threshold set at 30), as well as in (**m**) with *n* = 8. One-way ANOVA was used in (**c**) with triplicate repeats. All results mentioned above except (**l**) and (**m**) are presented with mean ± SD. (*P < 0.05, **P < 0.01)

Longitudinal analyses of vascular invasion of tumor cells in our metastatic mixed tumor model showed that VHL-KO cells invaded early into the circulation and their presence enhanced the subsequent escape of VHL-WT cells into the circulation (Supplementary Fig. 3c). These results suggested that VHL-KO cells could be influencing the vascular intravasation step.^45^ To study the intravasation process, we set up a 3D cell culture system that placed a layer of tumor cells above a layer of human umbilical vein endothelial cells (HUVEC), separated by a layer of Matrigel (Supplementary Fig. 4k). In this assay, the presence of the top layer of VHL-KO or 1:1 mixed cells, but not VHL-WT cells, induced significant destruction of endothelial cells (Supplementary Fig. 4k). A prior study showed that different types of tumor cells induced necroptosis in endothelial cells to promote intravasation, but RCC models were not investigated.^45^ Co-culturing with RC-VHL-WT or RC-VHL-KO cells with HUVEC cells did not activate the necroptosis markers MLKL or RIP in HUVECs (Fig. 6a, b). However, RC-VHL-KO cells induced robust apoptosis in HUVEC cells, as indicated by cleaved caspase 3 (Fig. 6a) and apoptosis reporter assays (Fig. 6c). Importantly, the anti-POSTN antibody blunted the HUVEC cell apoptosis (Fig. 6a, c). We further assessed endothelial destruction and vascular leakage in vivo in the CAM tumor system with the Miles assay. As shown in Fig. 6d, the vasculature of the mixed tumors was leakier than that of the VHL-WT tumors.

Given the multiple pro-metastatic influences exerted by POSTN, we deduced that blocking POSTN could suppress metastasis in vivo. The RC-VHL/POSTN-KO double-knockout cell line exhibited reduced promotion of VHL-WT cell motility in co-culture (Fig. 5f). Renal tumors containing 1:1 mixtures of VHL-WT cells and VHL/POSTN-KO cells showed no evidence of lung metastases compared to mixed tumors with VHL-WT and VHL-KO cells (Fig. 6e). Furthermore, treatment with MPC5B4 greatly suppressed lung metastases as assessed by gross lung morphology (Fig. 6f), lung weight (Fig. 6g), histological assessment by H&E (Fig. 6h) and IF staining (Fig. 6i). The POSTN-blocking treatment did not impact primary tumor growth significantly (Fig. 6j). We further assessed the anti-POSTN antibody treatment on the 1:1 mixed CAM tumors of case #22. Administration of MPC5B4 to the mixed tumors significantly reduced tumor cell dissemination into the chick embryo liver (Fig. 6k, l) without significant reduction in primary tumor growth (Fig. 6m).

**Fig. 7:**
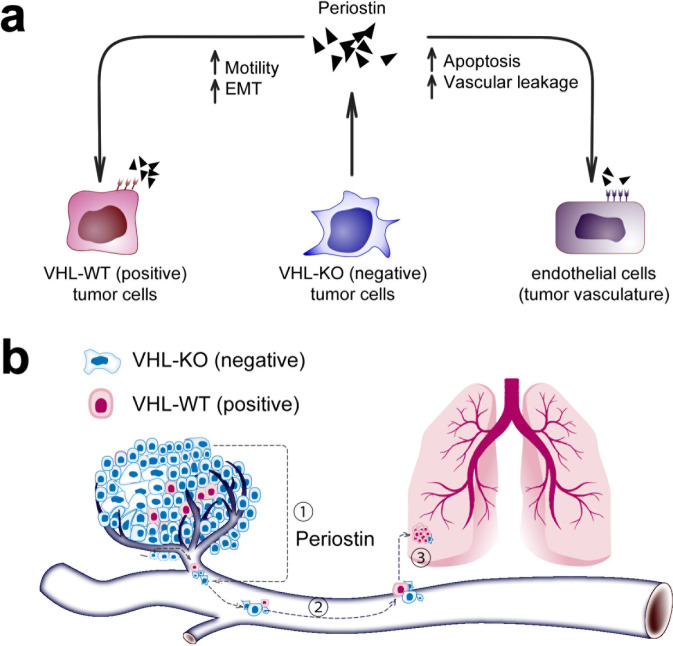
Paracrine action of POSTN in the collaborative metastasis model between VHL- and VHL + cells. **a** A summary of the pro-metastatic paracrine functions mediated by POSTN. **b** The cooperative metastatic mechanism uncovered paracrine-promoting interactions between VHL-KO and VHL-WT cells, mediated by POSTN, at the tumor proper and the intravasation step (1) of the metastatic cascade. The potential impact of the cooperative metastatic mechanism on downstream steps of the cascade, such as cell survival in the circulation (step 2), and metastatic colonization (step 3) requires further investigation

These data support POSTN as an important metastasis mediator secreted by VHL-KO cells that promotes the EMT and motility in VHL-WT cells, as well as the destruction of adjacent blood vessels (Fig. 7a). Thus, targeted blockade of POSTN appears to be a promising approach to inhibit the deadly metastatic process in ccRCC.

In this study, our VHL-deleted RCC models reveal a novel metastatic mechanism that relies on cooperative interactions between two distinct populations of tumor cells: VHL-KO (VHL^−^) and VHL-WT (VHL^+^) cells (Fig. 7b). VHL-KO cells displayed an EMT and highly motile phenotype, but grow poorly in vivo. However, VHL-KO cells induced an aggressive behavior in VHL-WT cells that promoted their fervent metastasis. Many of the metastatic phenotypes of our preclinical models were observed in clinical tumor specimens, including VHL loss resulting in the upregulation of HIF-1α and POSTN. These results lend credibility to a cooperative mechanism of metastasis operating in human ccRCC. This discovery of a novel cooperative metastatic mechanism and demonstration of the critical mediators of its crosstalk opens up novel therapeutic avenues to control ccRCC metastasis.

## Discussion

In RCC and other epithelial cancers, metastasis is the major cause of mortality. The complex nature of metastasis, coupled with an incomplete understanding of its mechanism, pose significant challenges to devising effective treatments. For the past three decades, the progression model has been the common, prevailing concept for how metastasis occurs.^46^ This model postulates that multiple progressive mutational events occur to enable a small fraction of cells to acquire full metastatic potential. Subsequent studies showed that clonal evolution and selection can enhance not only metastatic potential but also achieve metastatic site specificity.^47,48^ The cooperative model of metastasis uncovered here proposes a different mechanism in which active crosstalk between two populations of tumor cells is needed to achieve metastasis.

Our spontaneous metastatic RCC models documented the interactive cross communications between VHL-KO (VHL^−^) and VHL-WT (VHL^+^) cells. Intriguingly, EMT^+^ VHL-KO cells are themselves poorly proliferative, but drive the metastatic process by inducing aggressive behaviors, such as enhanced proliferation and motility, in the otherwise nonmetastatic VHL-WT cells. The loss of VHL upregulates HIF-1α, which in turn stimulates the transcription of *POSTN*. POSTN promotes metastasis by elevating EMT and motility in neighboring VHL-WT cells and also causes vascular destruction to facilitate the escape of tumor cells into the circulation. However, POSTN does not promote the proliferation of VHL-WT cells. Given that POSTN impacts multiple steps in the metastatic cascade, we went on to show that blocking its function by a monoclonal antibody halted lung metastasis in murine and human spontaneous metastasis models.

Prior studies have implicated pathways downstream of the VHL and HIF axis, such as CXCR4 and CYTIP or CDCP1 and FYN, being drivers of distant metastases in ccRCC.^49,50^ Our investigation does not support the metastasis-driving roles of these genes in our models (data not shown). The discrepancy could be due to different metastatic models used. POSTN’s mechanism of action in metastasis reported here is novel. It is a stromal protein that promotes integrin-dependent cell adhesion and motility during bone and cardiac development.^51,52^ The overexpression of *POSTN* is observed under EMT and hypoxia,^52,53^ the conditions of our VHL-KO cells. Periostin was reported to bridge the colonization of breast cancer cells to their terminal lung-metastatic site,^41^ placing its involvement at the distal end of the metastatic cascade. Our results suggest that POSTN acts at the tumor-proximal intravasation step. We have not fully investigated its role in the latter steps of metastasis. A POSTN neutralizing antibody was shown to block metastasis in an ovarian cancer model.^54^ In total, the clinical applicability of a POSTN-targeted therapy to block metastasis clearly warrants further investigation.

The collaborative interactions to promote metastasis between tumor and stromal cells, such as fibroblasts, pericytes, and macrophages, are well-documented.^55–57^ The cooperation between distinct populations of tumor cells to advance the disease is under recognized. In a breast cancer study,^58^ overexpression of EMT transcription factors activated Hedgehog/GLI signaling and promoted aggressive behavior in non-EMT cells in a paracrine manner. The cooperation between EMT and non-EMT cells reported is highly reminiscent of the crosstalk in our VHL-KO and VHL-WT model, but with POSTN as a functional mediator in our system.

The pro-metastatic interactions between tumor cell populations with distinct EMT status reported here could represent the functional consequence of the intratumoral heterogeneity, well-recognized in ccRCC.^59,60^ A growing volume of evidence supports that intratumoral heterogeneity is a tumor evolution process in neuroendocrine cancers, such as neuroendocrine prostate cancer (NEPC), acquired during treatment leading to therapeutic resistance and metastatic progression.^61,62^ Advanced genomic, epigenetic, and transcriptomic sequencing technologies and bioinformatics have been applied to profile tumor tissues to decipher pathways that govern cancer progression and metastasis.^56,57,61–63^ Despite the great depth of information acquired in studying human RCC tumors, untangling the relevant functional pathways remains very challenging. This is in part due to a shortage of preclinical models that could reproduce or validate the functional activity in vivo. Further, the current information was based largely on detailed genomic or transcriptomic analyses, while the functional activity is governed more directly by the protein expression and biochemical activity of a gene than its RNA expression. Extensive evidence shows discordance between RNA and protein expression in cells due to post-transcriptional effects.^64,65^ For VHL and HIFα-regulated pathways that involve protein stability and post-transcriptional regulations, relying solely on genomic and transcriptomic data to investigate metastatic signaling might be insufficient. It would be prudent to integrate protein expression with gene-expression analyses to gain a comprehensive view of the biology in tumor metastasis. In sum, the study reported here provides an alternate idea of how the complex task of metastasis can be achieved by a heterogeneous tumor. This cooperative model can guide the search for more effective treatments to block metastases and address a clearly unmet need in cancer research.

## Materials and methods

### Cells, plasmids, and reagents

The RENCA (RC) cell line was purchased from ATCC and was maintained in RPMI-1640 supplemented with 10% fetal bovine serum and 1 × penicillin/streptomycin (Thermo Fisher, CA, USA, catalog number: 15140122). All CRISPR/Cas9-mediated knockout RC cell lines were selected with puromycin and clonally purified via single-cell cloning in a 96-well plate. A lentiviral vector encoding HA-tagged mStrawberry (modified from pSicoR, Addgene, MA, USA, catalog number: 11579) was used to label RC-VHL-WT cells, while a vector with the same backbone encoding flag-tagged EGFP was used to label RC-VHL-KO, RC-VHL/HIF1A-KO, and RC-VHL/POSTN-KO cells. In addition, for in vivo studies, all cell lines were also marked with lentivirus expressing firefly luciferase to permit BLI. pGL3-basic was from Promega (CA, USA, catalog number: E1751) and was enzymatically digested with MluI and XhoI. The periostin promoter was cloned from the genomic DNA of RC cells with the following primers: forward – CGACGCGTTAAGGTGGACAGTGAGGAAGACACA, reverse – CCGCTCGAGTTGAGAAGAACGAGAGTAGAGATTTTAGG. The control *renilla* luciferase vector was pRL-TK from Promega (CA, USA, catalog number: E2231). The plasmid for overexpressing constitutively-active *HIF1A* was from Addgene (MA, USA, catalog number: 44028).

### Time-lapse microscopy for 2D scratch assay and 3D migration assay

A total of 1 × 10^5 tumor cells (e.g., 5 × 10^4^ cells each of VHL-WT and VHL-KO cells) were grown on a 24-well plate until reaching 90% confluence. The bottom of each well was scratched with the end of a 200 µl tip to form a gap. The cell migration was monitored continuously with a Nikon Eclipse Ti-E time-lapse microscope using a 10× objective, and a humidified, 37 °C environment containing 5% CO_2_. Specific fields of interest were set and recorded at 15 min intervals for 20 h using the FITC and TRITC channels. Nikon elements software was used to measure the migration speed of cells in each group.

Transwell chambers (0.4 μm pore size, Thermo Fisher, catalog number: CLS3470-48EA) were assembled in a 24-well plate. One milliliter of RPMI-1640 medium supplemented with 10% fetal bovine serum and 50 ng/mL EGF was added to the bottom chamber. HUVECs were seeded on the bottom of the Transwell chamber at a cell number of 1 × 10^5^. On day 2, a layer of Matrigel (Corning, catalog number: 356234) was coated on top of the layer of HUVECs and placed back in a 37 °C incubator to solidify: 100 µL for migration assay or 30 µL for 3D in vitro intravasation assay. Tumor cells (1 × 10^5^) were then seeded onto the top of the Matrigel. A Nikon Eclipse Ti-E time-lapse confocal microscope was used to image cell migration. The z-step parameters were set with the HUVEC cell layer as the bottom and the tumor cell layer as the top with approximately 200 stepwise stacks for scanning every 15 minutes for 48 h.

### CAM tumor xenograft model, renal tumor implantation, and anti-periostin treatment studies in mice

Establishment of CAM tumor xenografts and their analyses were performed as previously described.^32,66,67^ Intrarenal implantation of 1 × 10^6 total RC or AC tumor cells was performed as previously described.^28,35^ One week after implanting a 1:1 mixture of VHL-WT to VHL-KO cells, 10 mg/kg of MPC5B4 mAb was injected via tail vein three times per week for 4 weeks. The animals were imaged and sacrificed. Tissues were harvested, fixed, paraffin embedded, and cut for histological analyses. This study was approved by the UCLA Institutional Review Board.

### Isolation and cultivation of primary ccRCC tumor cells

With the consent of patients, primary ccRCC tumor samples were collected and chopped into pieces with sterile scissors and scalpels into RPMI-1640. Tissue chunks were transferred to a 15-mL conical tube and centrifuged at 300 × *g* at room temperature for 5 min. The supernatants were carefully discarded and the tissue pellet was resuspended in 2.6-mL prediluted 3 U/L Liberase TM (Sigma-Aldrich, catalog number: 5401119001) in RPMI-1640 media. The tube was placed on a100 RPM rotator at 37 °C for 1 h. When the tissue was fully digested and no chunks were visible, cells were centrifuged at 300 g at room temperature for 5 minutes. The pellet was further treated with prediluted 1× red blood cell lysis buffer (BD Biosciences, catalog number: 555899) in sterile water for 15 min and washed once with PBS. Cells were resuspended in RPMI-1640 supplemented with 10% fetal bovine serum and 1× penicillin/streptomycin and cultured in a humidified, 5% CO_2_ incubator at 37 °C.

### Human ccRCC patient specimens

The tissue microarray was constructed from a cohort of 357 patients who underwent nephrectomy for sporadic RCC at UCLA between 1989 and 2000, as previously described.^43^ Clinical data, including age, gender, Eastern Cooperative Oncology Group performance status, and pathologic data (including tumor-node-metastasis stage, histologic subtype, and Fuhrman grade) were collected for each case. This study was approved by the UCLA Institutional Review Board.

Large tumor tissues from primary tumors, locally invasive tumors, or metastases were obtained from 26 patients who underwent radical nephrectomy in the Department of Urology at the Ronald Regan Medical Center, UCLA, from 2015 to 2018. All patients provided informed consent before surgery, and all experiments were performed according to the approved guidelines, complying with the principles for the use of human tissues under the Declaration of Helsinki. This study was approved by the Institutional Review Board of UCLA, IRB Protocol #11-001363.

### Whole exome sequencing and data analysis from TCGA-KIRC database

Genomic DNA from 4 pieces of tumor chunks from patient #22 and the patient derived cell line were extracted and run whole exome sequencing (WES) at The Technology Center for Genomics & Bioinformatics (TCGB). Subsequently, WES data was aligned to human genome GRCh37 and processed following GATK v4.1.4.0 best practices.^68^ Mutation calls were made using Mutect2^69^ and known variants were annotated using VEP.^70^ Copy number calls were made using CNVkit v0.9.1^71^ with tumor samples matched to a neutral copy number in silico reference genome. Copy number events were mapped to the gene-level using the R package CNTools v1.38.0.^72^ Without sequenced normal samples to filter out germline SNPs, variant calls were filtered using COSMIC by only selecting mutations that were present in the database. For comparisons between matched samples, variants were further filtered to those with VEP impact rating above “LOW” and variant allele frequency (VAF) greater than 0.1.

While for the TCGA-KIRC dataset, Mutect2 MAF variant allele frequencies and SEG file copy number data for TCGA-KIRC samples were accessed through the Genomics Data Commons.^73^ Consensus estimates of tumor purity combining available measures from WES (ABSOLUTE), methylation profiling (LUMP), RNA sequencing (ESTIMATE), and IHC analysis were collected from the study of Aran D et al.^74^ and estimates of tumor ploidy based on ABSOLUTE from that of Hoadley KA et al.^75^ Samples were filtered for those where complete data was available and purity estimate greater than 0.4. Samples were also further filtered for those with reasonable agreement between ABSOLUTE and ESTIMATE purity estimates (difference less than 0.3), resulting in the removal of 21 samples. For VAF adjustment, adjusted frequencies between 1 and 1.1 were set to 1. Three samples with incompatible values within our equations was removed from analysis. Supplementary analysis using only ABSOLUTE estimates of tumor purity further reduced sample sizes since not all samples with consensus estimates of purity had contributing ABSOLUTE estimates and ABSOLUTE estimates more often (*n* = 17) led to incompatible values in our equations. Copy number and allele frequencies were adjusted to account for tumor purity and ploidy by assuming that the normal contamination is diploid, and using the following formulas:

$$CN_t = \frac{{2^{CNR} \ast \left( {2 \ast \left( {1 - PT_t} \right) + PL_t \ast PT_t} \right) - 2 \ast (1 - PT_t)}}{{PT_t}}$$

$$CNR_{adj} = \log _2\left(\frac{{CN_t}}{2}\right)$$

$$AF_{adj} = \frac{{VAF \ast (PT_t \ast CN_t + \left( {1 - PT_t} \right) \ast 2)}}{{PT_t \ast CN_t}}$$

CN_t_ = Tumor copy number PT_t_ = Tumor purity PL_t_ = Tumor ploidy CNR = Observed log2 copy number ratio CNR_adj_ = Purity and ploidy adjusted copy number ratio AF_adj_ = Purity and ploidy adjusted somatic variant allele frequency VAF = Observed variant allele frequency

### Single-cell sequencing and analysis

Two single cell sources were prepared for single-cell RNA sequencing in our study: the single cell nuclei from the frozen primary tumor tissue chunks, and the cryopreserved tumor cell suspension previously cultured overnight at passage 0 (P0) after disassociated from the same patient’s fresh tissue (#22 as shown in Table 1).

For frozen tissue chunks from patient #22, cell membranes were not able to be maintained intact after freeze-thaw cycle due to technological limit, but only the cell nuclei can be remained according to the ‘Frankenstein’ protocol maintained by 10× genomics company. The purpose to incorporate tissue derived cells in singe-cell sequencing is to explore the cell transcriptomics in their original status as hypoxia. This batch of cell nuclei were subjected to single-cell nuclei sequencing provided by the Technology Center for Genomics & Bioinformatics (TCGB) at UCLA.

Considering that VHL is expressed both in cytoplasm and nucleus, cryopreserved single cell suspension that’s been cultured for overnight after disassociation of fresh tissues from the same patient (#22) was used as a second input for single-cell sequencing. The digestion of fresh tissues were made by Liberase (Cat#5401119001, Sigma Aldrich, USA) at 13 u/ml in RPMI-1640 media at 37 °C cogwheel rotator. To make sure the majority of cells sequenced are tumor cells rather than normal cells, only attached cells were included to eliminate the normal blood cells as erythrocytes and white blood cells. Also, trypsin-EDTA selective detachment method was used to eliminate the stromal cells as fibroblast. RNA extraction and quality control were implemented by TCGB at UCLA on the Illumina NextSeq500 platform with single-end 1 × 75 base pair read length. Single-cell RNA sequencing results were demultiplexed and aligned to the prebuilt human reference genome GRCh38-2020-A annotated with transcript information from GENCODE v32 using the Cell Ranger v4.0.0 pipeline following all default parameters to generate count matrices.^76^

Single cell data was filtered to include cells with total counts greater than 2000, total number of expressed genes greater than 1000, and percentage of mitochondrial reads less than twenty percent. Unnormalized raw count data was input to scImpute v0.0.9 for identification of read dropout events and imputation.^77^ In total, 432 single cell nuclei from tissue chunks and 1343 intact single cells from frozen tissue cell suspensions were included in the analysis. Count matrices were then processed using the Seurat v4 workflow for data transformation and scaling, dimensionality reduction through uniform manifold approximation and projection (UMAP), and differential expression through the MAST package.^78–80^ Geneset enrichment analysis (GSEA) was then carried out using FGSEA v1.14.0 on a gene list pre-ranked with signed log10 p-values from the differential expression results.^81^ Genesets from the Hallmarks, KEGG, and Reactome databases accessed through the msigdbr v7.1.1 package were included in analyses.^82^

### Bulk RNA sequencing and analysis

RENCA and ACHN cells for bulk RNA sequencing were trypsinized and sent to the TCGB at UCLA for subsequent sample processing including RNA extraction and DNase treatment. Libraries were sequenced on the Illumina Hiseq3000 platform with single-end 1 × 50 base pair read length. Gene expression quantification from bulk RNA sequencing was generated using Salmon v1.2.1 run in mapping-based mode.^83^ Reads were selectively aligned to the GENCODE vM25 mouse reference transcriptome with corrections for sequence-specific and GC content biases. Gene count data were then processed using the DESeq2 v1.22.2 package for normalization and differential expression analysis.^84^ As with single cell data, GSEA was carried out using FGSEA v1.14.0 on pre-ranked gene lists. For RENCA models the signed log10 *p*-value was used for gene list ranking and for ACHN models the log2 fold change of gene CPMs were used where no *p*-value could be calculated.

### Identification of preserved gene expression signatures across RNA sequencing datasets

Concordant changes in gene expression between VHL + vs. VHL- cells in single-cell RNA sequencing, VHL + vs. VHL- ACHN models, and VHL + vs. VHL- RENCA models were identified by calculating the signal-to-noise ratio (squared mean divided by the sample variance) of log2 fold changes across the three comparisons for each gene. The top 200 genes based on this metric were then selected for clustering using Ward’s minimum variance method and plotting using the heatmap v1.0.12 package.^85^ A similar approach was then applied to the NES scores from the GSEA results from each comparison and the top 30 gene sets based on this metric were selected.

### RENCA cell line mutation profiling upon VHL knockout

Bulk RNA sequencing of RENCA cell line with VHL knockout and the control VHL-WT cells was made as mentioned previously. In order to examine the possible mutation brought by VHL knockout, the mutation analysis was made by rMATS-DVR as reported by Jinkai Wang et al.^86^ The result is listed in Dataset 1.

### Statistics

Each experiment was performed at least in triplicate unless otherwise stated. Data are presented as mean ± standard deviation (SD) unless stated otherwise. Significance was determined by a paired, Student’s *t*-test when there were two groups or by a one-way ANOVA when there were three or more groups (GraphPad Prism version 6.0). A *p*-value cutoff of 0.05 was used to establish significance.

## Supplementary information

Supplementary_Materials-clean version

Original uncropped WB films

Supplementary Movie S1

Supplementary Movie S2

Supplementary Movie S3

Supplementary Movie S4

Supplementary Movie S5

Supplementary Movie S6

Supplementary Movie S7

Dataset 1

## Data Availability

The data underlying this article are available in the article and in its online supplementary material. Also, the sequencing datasets underlying this article are available in NCBI SRA submission system with accession project # “PRJNA763575” and can be accessed by reviewers with the link below: https://dataview.ncbi.nlm.nih.gov/object/PRJNA763575?reviewer=q1e3jbt752iq8vuf1l5mgqs7o6.
