# Supplementary material for: Tumor heterogeneity in VHL drives metastasis in clear cell renal cell carcinoma
Source: Signal Transduct Target Ther. 2023 Apr 17;8:155. doi: 10.1038/s41392-023-01362-2 (PMC10110583; doi:10.1038/s41392-023-01362-2)
Supplement: Supplementary file 1 — Supplementary_Materials-clean version [file 41392_2023_1362_MOESM1_ESM.docx]

Supplementary Materials for

Tumor Heterogeneity in VHL Drives Metastasis in Clear Cell Renal Cell Carcinoma

Junhui Hu^1^, Ping Tan^3^, Moe Ishihara^1^, Nicholas A. Bayley^1^, Shiruyeh Schokrpur^4^, Jeremy G. Reynoso^5^, Yangjun Zhang^10^, Raymond J. Lim^2,1^, Camelia Dumitras^2,1^, Lu Yang^3^, Steven M. Dubinett^2,1,12^, Parmjit S. Jat^6^, Jacques Van Snick^7^, Jiaoti Huang^8^, Arnold I. Chin^11,12^, Robert M. Prins^1,5^, Thomas G. Graeber^1,12^, Hua Xu^9,10^, and Lily Wu^1,11,12^

Correspondence to: [lwu@mednet.ulca.edu](mailto:lwu@mednet.ulca.edu); [xu-hua@whu.edu.cn](mailto:xu-hua@whu.edu.cn)

**This PDF file includes:**

Materials and Methods

Figures. S1 to S4

Tables S1 to S2

Captions for Movies S1 to S7

Captions for Data S1

**Other Supplementary Materials for this manuscript include the following:**

Movies S1 to S7

Data S1

Materials and Methods

Cells, plasmids, and reagents

The wildtype VHL coding sequence was amplified from normal proximal renal tubule cells HK-2 by the following primers with EcoRV on each 5’ end: forward – AAACGGATATCATGCCCCGGAGGGCG, reverse – CGATCGATATCATCTCCCATCCGTTGATGTGC. The L169P variant is point mutated from the wildtype VHL above by relay PCR: forward 1 - AAACGGATATCATGCCCCGGAGGGCG, reverse 1 - CAGGCTTGACTGGGCTCCG; forward 2 - CGGAGCCCAGTCAAGCCTG, reverse 2 – CGATCGATATCATCTCCCATCCGTTGATGTGC. The vector backbone was from Addgene #65726, pLV-CMV-LoxP-DsRed-LoxP-eGFP.

Cell proliferation assay

Cell proliferation was measured using the MTS assay and direct cell counting. For both assays, cells in log phase were counted and seeded on day 0 at a density of 1000 cells per well onto a 96-well plate, or 500 cells per well onto a 384-well plate. For the MTS assay, cell numbers were evaluated every 24 hours on days 1, 2, 3, 4, 5, and 6 using the MTS kit (Promega, CA, USA, catalog number: G3582) and measured with a Multiskan MK3 microplate reader (Thermo, USA). For direct cell counting, an ImageXpress workstation was used to photograph each well of a 384-well plate and count the DAPI-stained cells.

IHC and IF staining

Slides were baked at 65 °C for 20 minutes and deparaffinized through three 10min incubations in xylene then rehydrated in stepwise dilutions of ethanol from 100% to 50% followed by water. Citrate buffer was used for antigen retrieval in a vegetable steamer for 25 minutes. Blocking used 1% BSA, and the following primary antibodies were incubated overnight at 4 °C: anti-VHL (1:200, Abcam, USA, catalog number: ab135576), anti-flag (1:200, eBioscience, USA, catalog number: 14-6681-82), anti-HA (1:200, Santa Cruz Biotechnology, USA, catalog number: sc805), and anti-Ki67 (1:200, Vector Laboratories, USA, catalog number: VP-RM04). After three 7-minute washes in TBST, slides were incubated with secondary antibody (goat-anti-rabbit, catalog number: 111-035-045; goat-anti-mouse, catalog number: 115-035-062; both from Jackson ImmunoResearch Laboratories, USA) at a 1:200 dilution. Slides were washed three times in TBST for 7 minutes each. For IHC, slides were incubated with DAB (Biocare Medical, USA, catalog number: DB801R) and counterstained with hematoxylin. For IF, slides were incubated with with FITC-conjugated TSA (Perkin Elmer, USA, catalog number: SAT701001EA) or CY3-conjugated TSA (Perkin Elmer, USA, catalog number: NEL744001KT). After TSA staining, Hoechst 33342 was added to the slides for nuclear staining, and slides were sealed with glycerol then scanned at UCLA’s Translational Pathology Core Laboratory (UCLA).

In addition, spatial quantification analyses for slide case #22 were performed using HALOTM Image Analysis program by Indica Labs (USA). An initial positive stain tissue marker analysis was conducted with proper nuclear segmentation and dye threshold intensities. Under HALOTM 3.0 Spatial Analysis Module, infiltration and density heat map algorithms were used to establish spatial relationships important for VHL-POSTN paracrine crosstalk.

Flow cytometry

Primary tumors and lungs of mice were dissected, minced into small pieces, and digested with 0.2% collagenous II at 37 °C on a 100 RPM shaker. The cell suspensions were passed through 70-µm cell strainers. The digested cells were stained with Hoechst 33342 for 15 minutes and analyzed by flow cytometry. Similarly, chicken and mouse blood were collected and lysed with red blood cell lysis buffer (BD Bioscience, USA, catalog number: 555899). Cells were then analyzed by flow cytometry for mStrawberry and EGFP expression. The gating was made by setting up control group of cells with vehicle control plasmid only that do not express EGFP or mStrawberry proteins. The Hoechst 33342 control cells were prepared by no dye treatment in the gating process.

qRT-PCR

Cell mRNAs are extracted with traditional Trizol (Cat#15596026, ThermoFisher, USA) method as mentioned in Shiruyeh Schokrpur et al ^1^, treated with DNase (Cat#18068015, ThermoFisher, USA) and reverse transcribed with PrimeScript 1st strand cDNA Synthesis Kit (Cat# 6110B, Takara, Japan). SensiFAST SYBR (Cat# BIO-98005, Bioline, USA) was used to for PCR reaction and signal read at Bio-Rad CFX96 Thermocycler. All primers used in this manuscript are listed in the Supplementary Table 1.

Miles Assay

Miles Assay is undertaken accordingly as noted in Diana Moughon et al ^2^

Promoter reporter assay

The validation of interaction of HIF1A and POSTN promoter was undertaken by the manual of Promega vectors pGL3-basic and pRL-TK. The vectors were cloned as described in the section of “Cells, plasmids and Reagents” and 293 cells were seed in a 24-well plate at 1×10^5/well on day 0. Then the pGL3-basic and pRL-TK plasmids were transfected into all wells, with HIF1A overexpressing plasmid in experimental group and its control plasmids in control group were transfected with FuGENE HD transfection agent (Cat#E2311, Promega). Upon 48 hours incubation, cells were lysed and measured by CLARIOstar Plus plate reader (BMG Labtech, USA).

VHL stability test

786-O cells were cultured and seeded in the 6-well plate at 5×10^5 cells per well on day 0. On day 1, each well was transfected with either 3000ng wildtype VHL or L169P variant plasmid with 9ul Promega FuGENE transfection reagent (cat# E2311, Promega, USA) followed with 24 hours incubation. Upon harvest, cycloheximide (CHX, 100μg/ml, Cat# 357420010, Acros Organics, USA) was added on each well and RIPA lysis buffer was used to harvest cells at 0, 3 and 6 hours upon CHX addition. The harvested protein was subjected to western blot analysis as mentioned earlier.

Western blot, necroptosis, and apoptosis reporter assay

For Western blot, 1×10^6 HUVECs were seeded onto the bottom of 6-well-plate Transwell chambers (1 μm pore size, Falcon, catalog number: 353102) with 1×10^6 tumor cells in the top chamber, with or without 1 μg/mL anti-POSTN MPC5B4 monoclonal antibody (mAb), with or without cilengitide in concentrations indicated in the figure legends. Cells were harvested after 48 hours for whole-cell-lysate protein extraction with RIPA buffer (ThermoFisher, catalog number: 89901) supplemented with proteinase inhibitors (Thermo Fisher, catalog number: 78430). Samples were then boiled for 10 minutes in 6× SDS loading buffer and loaded onto the 10% gels. Blots were probed with anti-phospho-RIP (Ser166)(1:1000), anti-RIP(1:1000), anti-phospho-MLKL (Ser358)(1:1000), anti-MLKL(1:1000), anti-caspase-3(1:1000), and anti-cleaved caspase-3(1:1000)from the Apoptosis/Necroptosis Antibody Sampler Kit (Cell Signaling Technology, MA, USA, catalog number: 92570). Blots were imaged and analyzed on a ChemiDoc XRS+ with associated Image Lab software (Bio-Rad).

For the necroptosis reporter assay, 24-well-plate Transwell chambers (0.4 μm pore size, Thermo Fisher, CA, USA, catalog number: CLS3470-48EA) were seeded with 1×10^5 HUVECs on the bottom and 1×10^5 tumor cells on the top of the chamber with or without 1µg/mL anti-POSTN MPC5B4 mAb. After 48 hours, HUVECs were washed with PBS once and a solution of 1.6 μM ethidium homodimer III (EthD-III, Biotium, CA, USA, catalog number: 400050) and 2 μM Hoechst33342 (Biotium, CA, USA, catalog number: 40045) were added to cells and incubated in a humidified, 5% CO_2_ incubator at 37 °C for 15 minutes. Microscope images were taken of five random fields of each well with a 10× objective in DAPI and TRITC channels and quantified with ImageJ.

For apoptosis evaluation, HUVECs were cultured in Transwell chambers as described above. After 48 hours, the plates were equilibrated at room temperature for 10 minutes, and 200 μL of Caspase-Glo 3/7 reagent (Promega, catalog number: G8090) was added to each well. After being placed on a shaker at 300-500 RPM for 30 seconds, the reaction was incubated at room temperature for 1 hour and then analyzed for luminescence with a Synergy HT microplate reader (BioTek).


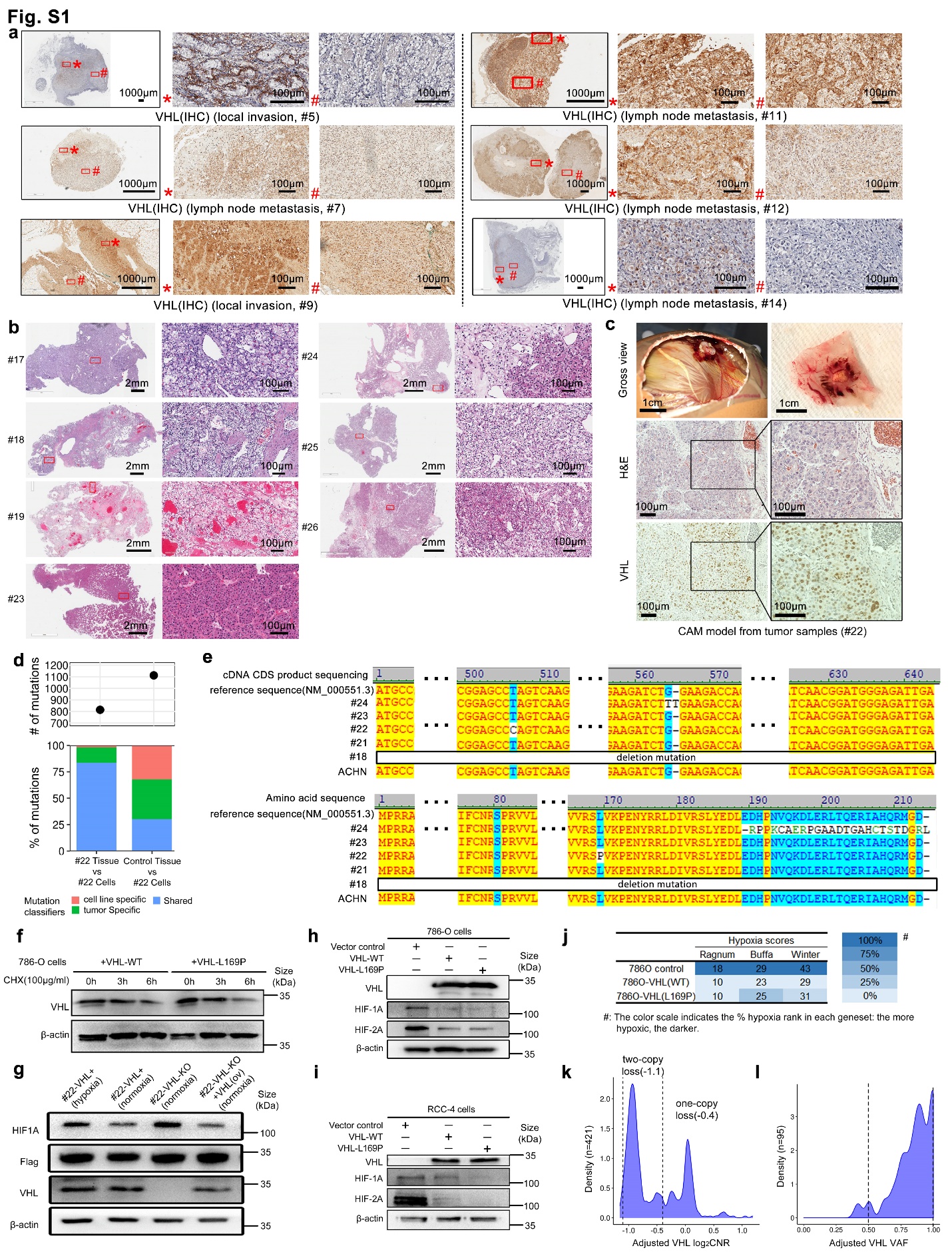
Figure. S1. Tumor histology, VHL gene sequences from specimen obtained from ccRCC patients, VHL stability test and hypoxia score assessment. a) Representative images of VHL IHC of case #5, 7, 9, 11, 12 and 14. Scale bar for the low magnification field: 1000μm; for the high magnification field: 100μm. b) H&E stains of tumor specimen from case #17, #18, #19, #23, #24, #25 and #26 (Table 1). Scale bar for the low magnification field: 2mm; for the high magnification field: 100μm. c) CAM tumors of case #22 were established by implanting small tumor chunks. Images of H&E stain and VHL IHC of the CAM tumor was shown. Scale bar for the gross view: 1cm; for the microscopic field: 100μm. d) Comparative genomic analysis of patient tumors derived cell line #22, with either the matched primary tumor tissue or control tissue from another ccRCC patient based on variant calls with an associated COSMIC ID. e) VHL gene sequences of ACHN, #18, #21, #22, #23 and #24 are shown. f) VHL stability test upon cycloheximide (100μg/ml) treatment for 0, 3 and 6 hours and the protein level were examined by western blot in 786-O cells, with either wildtype VHL or its L169P variant overexpression. g) Western blot analysis of VHL, flag and HIF1A in the primary cell line from patient tumor sample #22 in either hypoxia or normoxia, and VHL knocked out #22 cells with or without VHL artificial overexpression. Flag tag is transfected with wildtype HIF1A into cells as transfection control. h) Western blot analysis of VHL, HIF1A and HIF2A in 786-O cells with artificial overexpression of either control vector, wildtype VHL or L169P variant. i) Western blot analysis of VHL, HIF1A and HIF2A in RCC-4 cells with artificial overexpression of either control vector, wildtype VHL or L169P variant. j) hypoxia score assessment of 786-O cells with artificial overexpression of either control vector, wildtype VHL or L169P variant. k) Density plot showing the copy number ratios (CNRs) of the VHL locus in the TCGA-KIRC cohort (n = 421) after adjusting for both tumor purity and ploidy with estimates from ABSOLUTE. A CNR value of -1.1 represents a two copy loss and -0.4 indicates one copy loss of VHL (dotted line). l) Density plot showing the variant allele frequency (VAF) of somatic VHL mutations in the TCGA-KIRC cohort after adjusting for both tumor purity and ploidy with estimates from ABSOLUTE (n = 95 cases fitting analysis criteria as described in Materials and Methods).


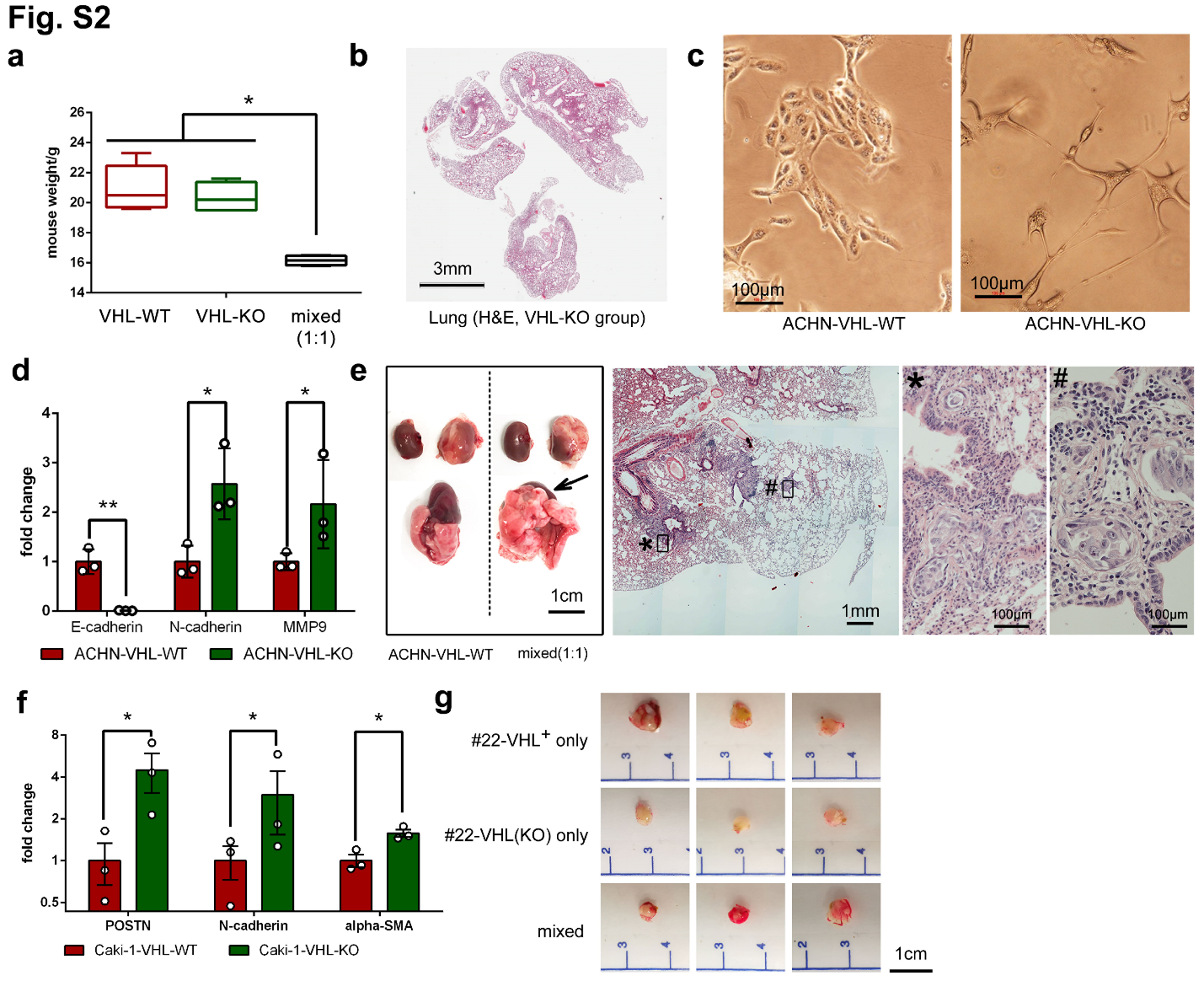
Figure. S2. Supplementary IHC staining and mouse weight from mice model, and the ACHN EMT changes upon VHL knockout and CAM tumor gross view from the patient #22 primary tumor derived cells. a) Body weight of mice bearing 1:1 mixed tumor was significantly less than those bearing VHL-WT or VHL-KO tumors at 4 weeks after tumor implantation. One-way ANOVA was used in the comparison with n=6 and presented with mean ± SD. b) H&E stain of lungs from a VHL-KO (clonal selected cells) tumor bearing mouse. Scale bar: 3mm. c) VHL-deleted ACHN human RCC line, AC-VHL-KO cells, display EMT cell morphology compared to the parental VHL+ ACHN (AC-VHL-WT) cells. Scale bar: 100μm. d) Gene expression assessed by RT-PCR showed elevated EMT markers in AC-VHL-KO cells. e) Gross assessment (scale bar: 1cm) of tumor growth in the kidneys and lungs of nude mice receiving AC-VHL-WT or a 1:1 mixture of AC-VHL-WT and AC-VHL-KO cells. H&E staining of the lung from the mixed implanted group are shown in the right panel. Scale bar for the low magnification field: 1mm; for the high magnification field: 100μm. f) PCR analysis of POSTN, N-Cadherin and alpha-SMA in VHL wildtype cell line Caki-1 upon CRISPR/Cas9 mediated VHL knockout. Student t-test was used in the comparisons (d) and (f) with triplicate repeats and presented with mean ± SD. g) Gross view of the CAM tumors grown from either the original primary cancer cell line of patient #22 that’s VHL+, the derived VHL-KO cell line or the 1:1 mixture of both. Scale bar: 1cm. (*: p<0.05, **: p<0.01)

**
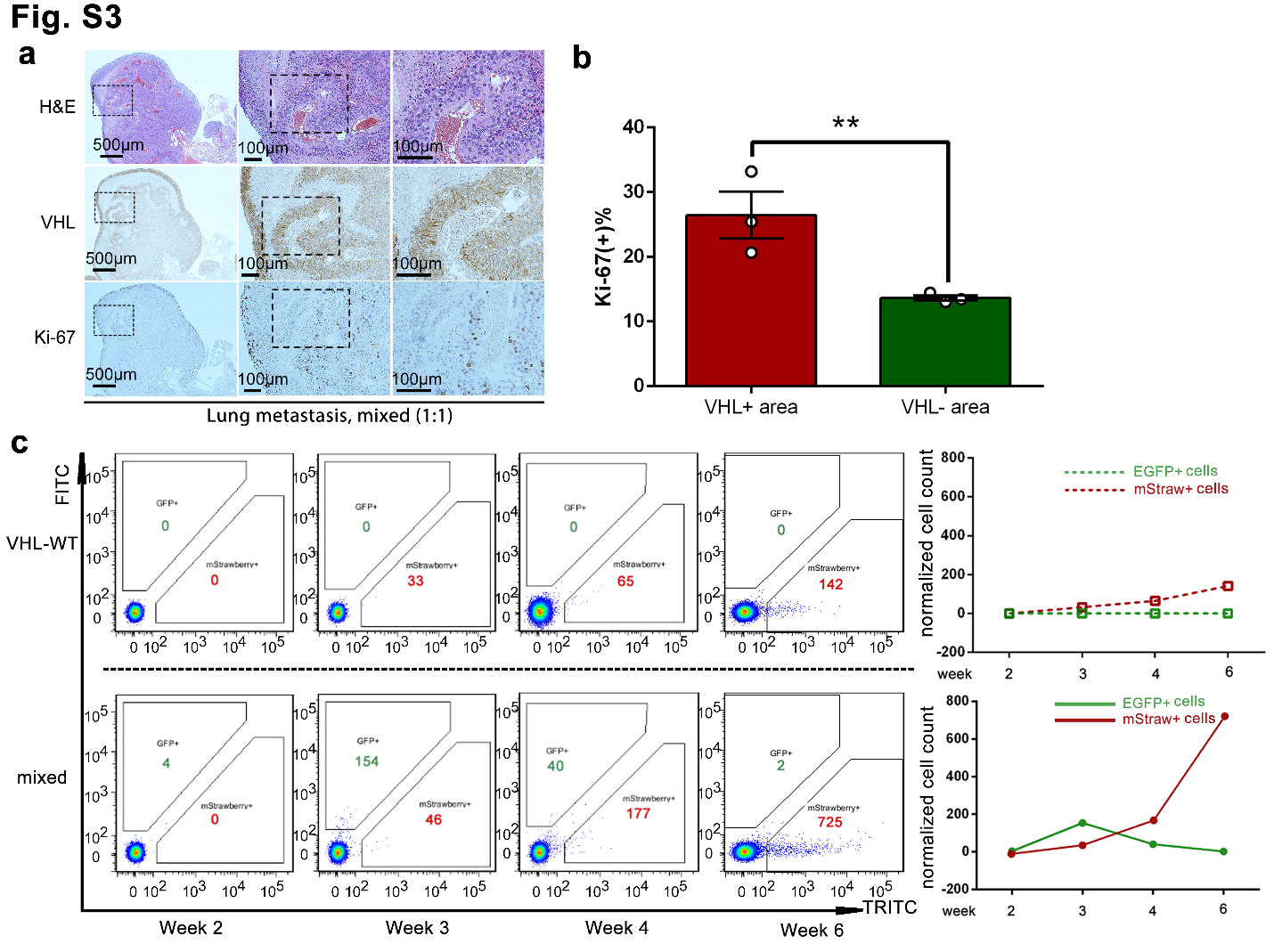
Figure. S3. Temporally sequential sampling of blood in analysis of CTC constituents and supplementary IHC staining of Ki-67 in the lung metastasis of RENCA mouse model.** a) IHC staining of VHL and Ki-67 in lung metastasis of mouse model which receives 1:1 mixture of VHL-WT RENCA cells and VHL-KO cells, as well as the quantification of Ki-67+ cell percentage in VHL+ and VHL- areas. Scale bar for the low magnification field: 500μm; for the high magnification field: 100μm. b) Quantification of Ki-67+ cells in either VHL+ cells area or VHL- areas shown in (a). Student t-test was used in the comparison with triplicate repeats and presented with mean ± SD. c) Sequential flow cytometric analysis of circulatory tumor cells at 2, 3, 4 and 6 weeks after implantation of VHL-WT or 1:1 mixed cells. (**: p<0.01)

**
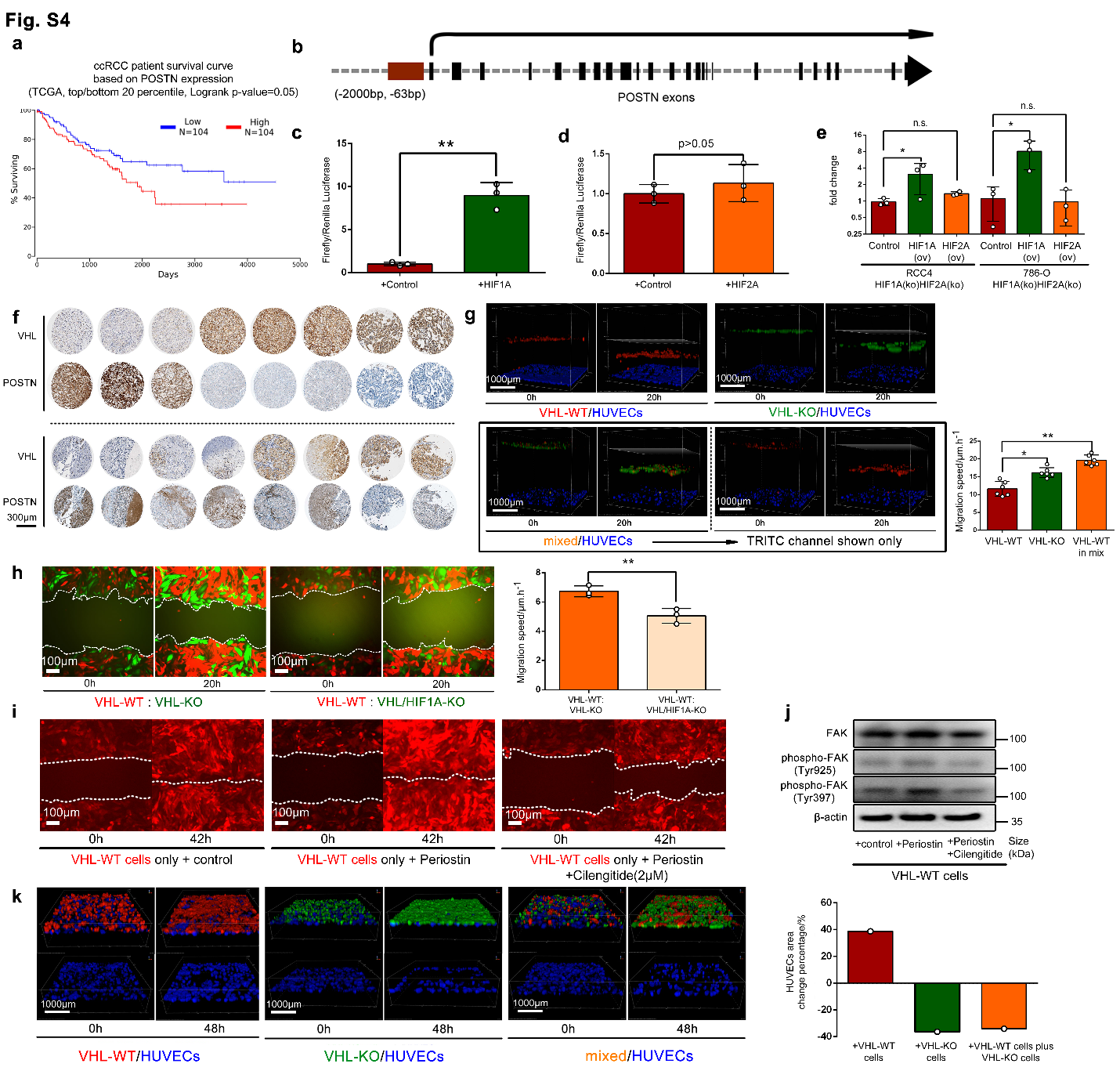
Figure S4. POSTN expression, regulation and tumor cell/endothelial cells interaction assessment. a)** According to TCGA ccRCC database KIRC, the patient survival curve is produced with the top and bottom 20% in POSTN expression from the http://www.oncolnc.org website. **b)** Schematic illustration of the gene POSTN and the cloned promoter region at (-2000bp, -63bp) at pGL3-basic vector for promoter activity analysis in (c) and (d). **c)** The overexpression of constitutively active human HIF1A can significantly stimulate the promoter activity in pGL3-basic vector with phRL-TK vector as control in 293T cells. **d)** The overexpression of constitutively active human HIF2A does not alter the promoter activity in pGL3-basic vector with phRL-TK vector as control in 293T cells. **e)** On the HIF1A and HIF2A knockout RCC4 and 786-O cells, constitutively active human HIF1A but not HIF2A can enhance the level of POSTN as reflected by qRT-PCR. **f)** Tissue microarray (TMA) of over 300 cases of RCC patients were assessed for VHL and POSTN expression. Representative images from 16 cases showing inverse correlated expression pattern between VHL and POSTN. Scale bar: 300μm. **g)** RENCA VHL-WT cells (red), VHL-KO cells (green) or the mixture of both were observed to migrate through the matrigel layer (~2mm) from the top to the bottom, which was labeled as HUVECs cells (blue). The migrated distance of red cells (top left), green cells (top right), and red cells in mixture (bottom left) were quantified and analyzed (right). The video can be found in Supplementary Movie S2. Scale bar: 1000μm. **h)** Scratch assay in coculture of either RENCA-VHL-KO or RENCA-VHL(KO)HIF1A(KO) cells with RENCA-VHL-WT cells over 20 hours. The promoted motility of RENCA-VHL-WT cells was abrogated by additional HIF1A knockout in RENCA-VHL-KO cells. The video can be found in Supplementary Movie S4. Scale bar: 100μm. **i)** The addition of recombinant POSTN protein enhance the motility of VHL-WT cells and cilengitide inhibits this POSTN mediated effect. Scale bar: 100μm. **j)** Western blot of phospho-Tyr 397, phosphor-Tyr925 and total FAK in VHL-WT cells cultured alone, with the addition of recombinant POSTN protein and with the further addition of the integrin inhibitor cilengitide. β-actin was used as a loading control. **k)** A 3D endothelial invasion assay was performed by placing a layer of tumor cells (either mStrawberry-marked VHL-WT cells, EGFP-marked VHL-KO cells, or a 1:1 mixture of the two cell types) over a layer of Matrigel (~ 30μm thick) that was above a HUVEC endothelial cell layer (marked with tagBFP). Scale bar: 1000μm. HUVEC area size was assessed 48 hours after coculture (graph, right). Student t-test was used in (c), (d) and (h), and one-way ANOVA was used in (e) and (g) with triplicate repeats. All are presented with mean ± SD. (*: p < 0.05, **: p < 0.01)

Table S1.

Primer Sequences

| Primer Name | strand | Sequence (5'-3') |
| --- | --- | --- |
| Human-E-cadherin | forward | CGAGAGCTACACGTTCACGG |
|  | reverse | GGGTGTCGAGGGAAAAATAGG |
| Human-N-Cadherin | forward | TCAGGCGTCTGTAGAGGCTT |
|  | reverse | ATGCACATCCTTCGATAAGACTG |
| Human-MMP9 | forward | TGTACCGCTATGGTTACACTCG |
|  | reverse | GGCAGGGACAGTTGCTTCT |
| Human-HIF1A | Forward | ATCCATGTGACCATGAGGAAATG |
|  | Reverse | TCGGCTAGTTAGGGTACACTTC |
| Human-HIF2A | Forward | TTGCTCTGAAAACGAGTCCGA |
|  | Reverse | GGTCACCACGGCAATGAAAC |
| Mouse-E-cadherin | forward | CAGGTCTCCTCATGGCTTTGC |
|  | reverse | CTTCCGAAAAGAAGGCTGTCC |
| Mouse-N-Cadherin | forward | AGCGCAGTCTTACCGAAGG |
|  | reverse | TCGCTGCTTTCATACTGAACTTT |
| Mouse-MMP9 | forward | CTGGACAGCCAGACACTAAAG |
|  | reverse | CTCGCGGCAAGTCTTCAGAG |
| Mouse-alpha-SMA | forward | GTCCCAGACATCAGGGAGTAA |
|  | reverse | TCGGATACTTCAGCGTCAGGA |
| Mouse Periostin | Forward | CACGGCATGGTTATTCCTTCA |
|  | reverse | TCAGGACACGGTCAATGACAT |

Table S2.

Heatmap gene list (not in the same order as shown in Figure 2H)

|  | scRNAseq | RENCA | ACHN |
| --- | --- | --- | --- |
| LTN1 | 0.029949671 | 0.216259635 | -0.244046986 |
| SPRY2 | -0.004380649 | 0.459421787 | 0.802698714 |
| USP16 | -0.000114495 | 0.235033035 | 0.000230538 |
| TSC22D1 | -0.025668863 | 0.54509172 | -0.41371657 |
| LHFPL6 | -0.017287333 | 1.340616172 | -0.003494214 |
| APP | 0.020125674 | 0.453180406 | -0.091447121 |
| LACC1 | -0.017968804 | 0.296739654 | 0 |
| SCAF4 | -0.067969505 | 0.21401484 | 0.159967507 |
| GATD3B | 0.007226167 | -0.177075597 | -0.154909609 |
| PI4KA | 0.012392537 | 0.047625758 | -0.362028499 |
| PHF11 | -0.036077527 | 0.016611075 | 0 |
| CRYL1 | 0.102073523 | -0.175206023 | -1.372718193 |
| ELF1 | 0.01428615 | 0.193623564 | -0.351503747 |
| TGDS | -0.031250404 | 0.240657204 | -0.719228493 |
| PSMG2 | 0.018758002 | 0.132350191 | 0 |
| PSPC1 | 0.006439259 | 0.153276785 | 0.071533313 |
| NDUFV2 | 0.011319559 | -0.063061321 | -0.066775978 |
| GTF3A | 0.005442852 | 0.301256151 | 0.240531924 |
| DNAJC15 | 0.006295613 | -0.04245555 | 0 |
| CAB39L | 0.006307857 | -0.921437957 | -0.549919512 |
| MPHOSPH8 | 0.008606336 | -0.105851842 | 0.307111528 |
| FHOD3 | 0.086128862 | -0.126003949 | -0.253258758 |
| IL17RA | -0.003194394 | 0.149905586 | -0.366139862 |
| KATNAL1 | -0.045523327 | 0.064094925 | 0.656973322 |
| CHMP1B | 0.012205839 | -0.241737868 | 0.038834937 |
| KPNA3 | -0.034370674 | 0.383123218 | 0.341337115 |
| TMEM50B | -0.042203235 | -0.938410916 | 1.293829406 |
| B3GLCT | -0.002101911 | -0.108875852 | 0 |
| PAN3 | -0.02087726 | -0.111313071 | 0.411993587 |
| METTL4 | -0.036433048 | 0.024693983 | 0 |
| GART | 0.019316566 | 0.414009023 | -0.334226397 |
| THOC1 | -0.091948675 | 0.126644198 | 0.224440511 |
| CCT8 | 0.021592092 | 0.371413436 | 0.014382988 |
| SAP18 | 0.038138622 | 0.703984565 | 0.19489591 |
| TBC1D4 | -0.037566604 | 0.533558478 | -0.285812074 |
| USP14 | 0.038433147 | 0.380810136 | 0.112531799 |
| UFM1 | 0.014382199 | -0.118102997 | 1.141193957 |
| CCDC122 | -0.01402147 | 0.066287014 | -0.107584259 |
| PIK3C3 | 0.023648788 | -0.071157384 | 0.968983566 |
| PROSER1 | -0.045532276 | 0.182970939 | 0 |
| DONSON | -0.053238758 | -0.057225447 | -0.614444565 |
| RAB31 | 0.043382136 | -0.337296545 | -0.564208472 |
| SKA3 | -0.079767763 | 0.253112291 | 1.726455568 |
| MIPEP | 0.001046048 | 0.009989601 | 0.159097062 |
| GTF2F2 | -0.008380017 | 0.223072236 | -0.363829322 |
| RWDD2B | -0.015866189 | 0.05090644 | 0.570753568 |
| MED15 | -0.026637747 | 0.248915471 | 0.490193338 |
| MAPRE2 | -0.020010785 | -0.373551137 | -0.034813529 |
| PIBF1 | -0.01490406 | 0.118917244 | 0.175726102 |
| KLF5 | -0.021061328 | 1.830117243 | -0.222424082 |
| URB1 | -0.001845171 | 0.338767946 | 0.977628348 |
| FNDC3A | 0.034920668 | -0.008311107 | 0.053670049 |
| SETDB2 | -0.013869473 | 0.175070266 | -0.096796598 |
| YES1 | -0.065163816 | -0.080281182 | -0.129600015 |
| SLC5A3 | -0.026163414 | -2.232188386 | -0.349568779 |
| CHAF1B | -0.000817915 | -0.070582393 | 0.295882386 |
| MRPS6 | 0.023990202 | -1.735474351 | 0 |
| MPPE1 | 0.020551726 | -1.236698169 | 0 |
| OSBPL1A | -0.029964436 | -0.44073452 | 0.671476588 |
| HMGB1 | -0.302285567 | 0.244773558 | 0.09192867 |
| MZT1 | -0.088049752 | 0.631891994 | 0.095648525 |
| MRPL57 | 0.054301504 | 0.091382351 | 0 |
| SLC25A15 | 0.000934026 | -0.201261873 | -0.0559697 |
| IMPA2 | 0.114518842 | 0.387779653 | -1.354578711 |
| PARP4 | 0.035222289 | -0.012456644 | -0.590246179 |
| CXADR | -0.033936551 | 2.018312006 | 0 |
| HSPH1 | -0.118640371 | -0.017372665 | 0.136204702 |
| NDC80 | -0.085217579 | -0.213932782 | -0.190186725 |
| LNX2 | -0.006647531 | 0.143269871 | 1.687354294 |
| NUFIP1 | 0.032260082 | 0.900531899 | -0.014557074 |
| USPL1 | 0.003028246 | 0.409316793 | -0.745908307 |
| POMP | -0.056626815 | 0.063782558 | -0.257457034 |
| MTCL1 | 0.069080427 | 0.040189104 | -0.006771509 |
| OPTN | 0.058246493 | -0.286353213 | 0.314241192 |
| ATP9A | 0.030520607 | -0.382499106 | -0.360694172 |
| IFT27 | 0.01593635 | -0.200273005 | 0 |
| PISD | 0.035302873 | 0.202793403 | 0.086844983 |
| DYNLT3 | -0.010301747 | 0.104507568 | 0.105123037 |
| TTLL12 | 0.000606516 | 0.494949508 | -0.129384807 |
| PTGIS | 0.04413237 | 1.286564677 | -1.323248724 |
| STK11 | 0.000837313 | -0.06489724 | 0.414331162 |
| ATP9B | -0.004919215 | -0.408479404 | -1.536532809 |
| RNF114 | 0.030950793 | -0.055121537 | -0.101298783 |
| STARD13 | -0.012802636 | 0.151978771 | -0.691067961 |
| DSTN | -0.032200994 | 1.334089192 | 0.389005549 |
| POLRMT | -0.03459413 | -0.070900741 | 0.288465817 |
| SKA1 | -0.030812004 | 0.087538865 | 0 |
| AK3 | 0.011581844 | -0.594495471 | 0.393571862 |
| POLR1D | 0.019320043 | 0.298258553 | -0.235655755 |
| TXN2 | 0.032061323 | 0.060051749 | 0 |
| SLC39A6 | 0.028440924 | 0.072617286 | 0.393960356 |
| ETS2 | -0.038523198 | 0.628839114 | -0.546124846 |
| MAP1LC3A | 0.026276283 | -0.787277806 | -2.135865669 |
| EIF3D | 0.071123433 | 0.19224557 | 0.300966782 |
| SEPHS1 | -0.049493719 | -0.004831018 | -0.419477032 |
| PSTPIP2 | -0.010071475 | -0.227368408 | -1.511074527 |
| WDR13 | 0.02497332 | -0.415008232 | 0.427170804 |
| IPO5 | -0.052246651 | 0.870116625 | 0.048368778 |
| PSMG3 | -0.038182534 | 0.170877263 | -0.170889936 |
| TUBGCP3 | -0.018170643 | -0.010185271 | -0.274447085 |
| ZYX | 0.061664646 | 0.391704367 | 0.245784731 |
| NSF | 0.005868079 | 0.028397755 | 0.040950567 |
| PLXNB2 | 0.095441947 | 0.351964923 | 0.137083775 |
| CHD1 | 0.039036447 | 0.294164248 | 0.294818565 |
| LRRFIP2 | 0.014007181 | 0.074008514 | 0.09999971 |
| EIF4E2 | 0.039815244 | 0.197870123 | 0.102019734 |
| SIDT2 | 0.019061135 | 0.050377129 | 0.014322314 |
| SPOP | 0.048758886 | 0.032339381 | 0.121040421 |
| MEA1 | 0.05498862 | 0.113308498 | 0.240451561 |
| DDB1 | 0.036691685 | 0.262748754 | 0.213653803 |
| LIAS | 0.003125193 | 0.020250712 | 0.022734705 |
| SGMS2 | 0.037401766 | 0.229023413 | 0.268105505 |
| WAC | 0.047529237 | 0.291516643 | 0.192524675 |
| GTF2F1 | 0.043209905 | 0.304243911 | 0.285369277 |
| MELTF | 0.062156212 | 0.075092078 | 0.010871311 |
| ELAVL1 | 0.044303355 | 0.171200722 | 0.260656631 |
| MITF | 0.093987613 | 0.070608557 | 0.241661302 |
| PHAX | 0.02409906 | 0.150337049 | 0.164059907 |
| ABCF1 | 0.041824951 | 0.269207719 | 0.275590927 |
| SRA1 | 0.057506695 | 0.247136151 | 0.349428284 |
| DLG5 | 0.016372379 | 0.105817992 | 0.089408936 |
| MAPRE1 | 0.066312384 | 0.029376189 | 0.017623718 |
| CWC15 | 0.049734096 | 0.052263104 | 0.14781658 |
| VEGFA | 0.067477297 | 0.381506867 | 0.42800118 |
| CEBPZ | 0.054341186 | 0.335826356 | 0.322287243 |
| CTNND1 | 0.063080571 | 0.23188091 | 0.102248541 |
| PRKCA | 0.032699701 | 0.161905441 | 0.096375354 |
| RNF7 | 0.046409826 | 0.007763954 | 0.045514976 |
| GRHPR | 0.182021928 | 0.873533652 | 0.512538935 |
| ZNF451 | 0.028741342 | 0.137562267 | 0.170423932 |
| UBXN1 | 0.083457635 | 0.265186675 | 0.104640836 |
| QTRT1 | 0.035486474 | 0.18310564 | 0.206625055 |
| YTHDC1 | 0.037157295 | 0.179649726 | 0.215160565 |
| FAM53C | 0.030435855 | 0.169505611 | 0.129422907 |
| FOXP4 | 0.024907792 | 0.075200864 | 0.120192131 |
| FAM168A | 0.020631603 | 0.049773332 | 0.01539255 |
| BICD2 | 0.056518981 | 0.280995504 | 0.188596153 |
| CHMP2A | 0.071290475 | 0.013636599 | 0.052265177 |
| SLC6A8 | 0.076826585 | 0.022995846 | 0.034017516 |
| KIAA0100 | 0.024849426 | 0.131478747 | 0.115821976 |
| NOP53 | 0.188120185 | 0.678050069 | 0.327649165 |
| MMUT | 0.01151153 | 0.060533491 | 0.052965193 |
| RTCB | 0.034768872 | 0.126102195 | 0.172455892 |
| RNMT | 0.043763414 | 0.16067524 | 0.217422187 |
| LSM14A | 0.029632884 | 0.141082028 | 0.097459538 |
| ZFR | 0.032353834 | 0.14825829 | 0.16589233 |
| EIF3E | 0.087972002 | 0.189681943 | 0.346779061 |
| LCOR | 0.028355703 | 0.136193579 | 0.102804615 |
| TIPARP | 0.046876458 | 0.229477434 | 0.187039825 |
| RNF25 | 0.036801002 | 0.105022079 | 0.042153524 |
| PEA15 | 0.076063819 | 0.25384946 | 0.353530737 |
| EIF3D | 0.071123433 | 0.19224557 | 0.300966782 |
| SGF29 | 0.036884819 | 0.079504742 | 0.025197034 |
| CUL3 | 0.032338299 | 0.035917939 | 0.088430796 |
| UBE2Z | 0.072975404 | 0.32790456 | 0.272318335 |
| TCF25 | 0.066245006 | 0.264367131 | 0.288911709 |
| SPIN1 | 0.046342078 | 0.193753197 | 0.195023869 |
| BMP1 | 0.047506301 | 0.121980115 | 0.185223497 |
| ARL3 | 0.048170409 | 0.181844799 | 0.200028075 |
| FNDC3B | 0.051091151 | 0.06252387 | 0.141106478 |
| PBX2 | 0.030690962 | 0.119354763 | 0.124807376 |
| MCFD2 | 0.054724283 | 0.149325671 | 0.212897167 |
| CTDNEP1 | 0.048709141 | 0.155010295 | 0.083191701 |
| ZNHIT1 | 0.093977074 | 0.365270317 | 0.33859492 |
| AP3S1 | 0.071586615 | 0.035127308 | 0.118492463 |
| DDX56 | 0.084614638 | 0.314518089 | 0.233251445 |
| RBM42 | 0.028092162 | 0.038728363 | 0.07839812 |
| ELOB | 0.087350588 | 0.089744889 | 0.210956157 |
| DCUN1D3 | 0.062547767 | 0.226162975 | 0.21505998 |
| RAI14 | 0.078718376 | 0.151306868 | 0.052611436 |
| SF3B2 | 0.035837957 | 0.130626631 | 0.10765477 |
| FRA10AC1 | 0.01310695 | 0.017008753 | 0.004811964 |
| MRPL32 | 0.059571676 | 0.131380566 | 0.055328524 |
| TAF12 | 0.044857257 | 0.025270012 | 0.075305165 |
| TOPORS | 0.035122195 | 0.027249181 | 0.010533879 |
| TIMM9 | 0.075395647 | 0.169022097 | 0.239823293 |
| UBE3B | 0.04056258 | 0.100712553 | 0.051117955 |
| CCDC80 | 0.081317772 | 0.239713027 | 0.248267945 |
| PAIP1 | 0.051585503 | 0.110078185 | 0.153910641 |
| RAB18 | 0.052932915 | 0.095047031 | 0.147604042 |
| LASP1 | 0.050356937 | 0.08102761 | 0.13250203 |
| MAGED1 | 0.067617595 | 0.174843037 | 0.105170605 |
| PSMB5 | 0.097398537 | 0.187739348 | 0.086962362 |
| GLIS3 | 0.077883544 | 0.100919912 | 0.175517723 |
| EHMT1 | 0.020652862 | 0.051953147 | 0.036512383 |
| PSMC1 | 0.045322427 | 0.107758381 | 0.112950193 |
| TRIAP1 | 0.054019576 | 0.066333201 | 0.117375465 |
| CIAO1 | 0.057523479 | 0.027515009 | 0.06966866 |
| EIF3H | 0.097278737 | 0.23675541 | 0.189348184 |
| PSMB4 | 0.069200873 | 0.113932044 | 0.159279194 |
| NUTF2 | 0.059853882 | 0.132772729 | 0.13577629 |
| BTF3 | 0.047689249 | 0.058799547 | 0.097668286 |
| PITPNB | 0.038212874 | 0.054338099 | 0.080562757 |
| NMT1 | 0.05355703 | 0.061573568 | 0.029671073 |
| NCOA4 | 0.085565125 | 0.17025605 | 0.156753226 |
| RPRD2 | 0.086748757 | 0.049587284 | 0.088567295 |
| RPF2 | 0.072007906 | 0.057843848 | 0.101713535 |
| CAV2 | 0.110731122 | 0.183301521 | 0.167618168 |
| JUND | 0.141582858 | 0.176186811 | 0.205387289 |
| PPP4R3B | 0.043474107 | 0.052805942 | 0.049071585 |

**Supplementary Movies (separate files)**

Movie S1. 2D Scratch assay for RC-VHL-WT cells alone, RC-VHL-KO cells alone, or mixture of both. a) A scratch assay of RC-VHL-WT cells; b) A scratch assay of RC-VHL-KO cells; c) A scratch assay of mixture of both cells in a 1:1 ratio; d) The TRITC channel of scratch assay of the mixture of both cells in 1:1 ratio.

Movie S2. 3D Scratch assay for RC-VHL-WT cells alone, RC-VHL-KO cells alone, or mixture of both. a) A scratch assay of RC-VHL-WT cells; b) A scratch assay of RC-VHL-KO cells; c) A scratch assay of mixture of both cells in a 1:1 ratio; d) The TRITC channel of scratch assay of the mixture of both cells in 1:1 ratio.

Movie S3. 2D Scratch assay for RC-VHL-WT cells supplemented with fresh media (a) or the conditioned media (b) from RC-VHL-KO cells.

Movie S4. 2D Scratch assay for RC-VHL-WT cells in mixture with RC-VHL-KO cells (a) or with a compound deletion of VHL and HIF1A cells (b).

Movie S5. 2D Scratch assay for RC-VHL-WT cells in mixture with RC-VHL-KO cells (a) or with a compound deletion of VHL and POSTN cells (b).

Movie S6. 2D Scratch assay for RC-VHL-WT cells in mixture with RC-VHL-KO cells under the effect of control reagent PBS (a) or anti-periostin MPC4B5 mAb (b).

Movie S7. 2D Scratch assay for RC-VHL-WT cells in mixture with RC-VHL-KO cells supplemented with cilengitide at 0 (a), 2μM (b), 5μM(c) and 10μM (d).

Data S1. (separate file)

RENCA mutation profile upon VHL knockout

References

1 Schokrpur, S. *et al.* CRISPR-Mediated VHL Knockout Generates an Improved Model for Metastatic Renal Cell Carcinoma. *Sci. Rep.* **6**, 29032, (2016).

2 Moughon, D. L. *et al.* Macrophage Blockade Using CSF1R Inhibitors Reverses the Vascular Leakage Underlying Malignant Ascites in Late-Stage Epithelial Ovarian Cancer. *Cancer Res.* **75**, 4742-4752, (2015).
