# Supplementary figures and images for: Tumor heterogeneity in VHL drives metastasis in clear cell renal cell carcinoma
Source: Signal Transduct Target Ther. 2023 Apr 17;8:155. doi: 10.1038/s41392-023-01362-2 (PMC10110583; doi:10.1038/s41392-023-01362-2)

Fig. 4a

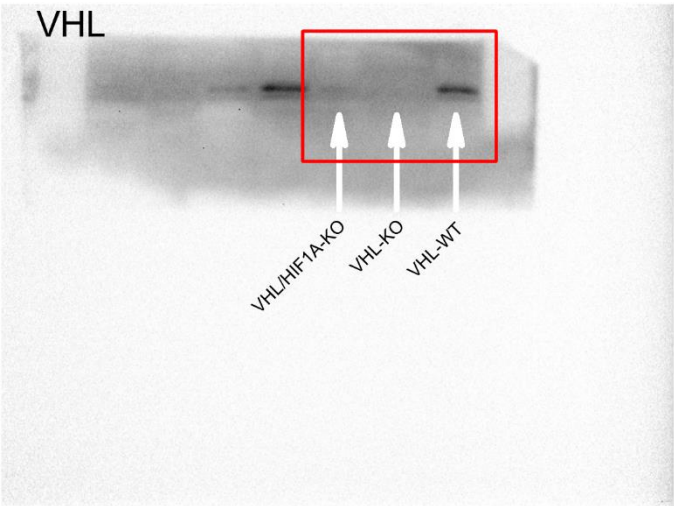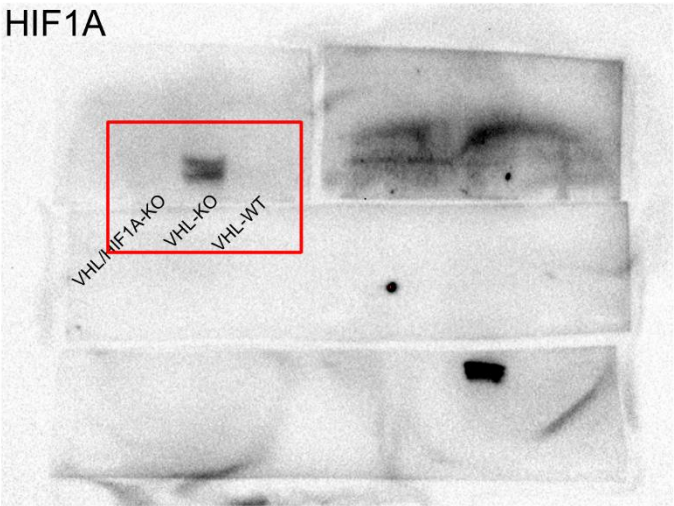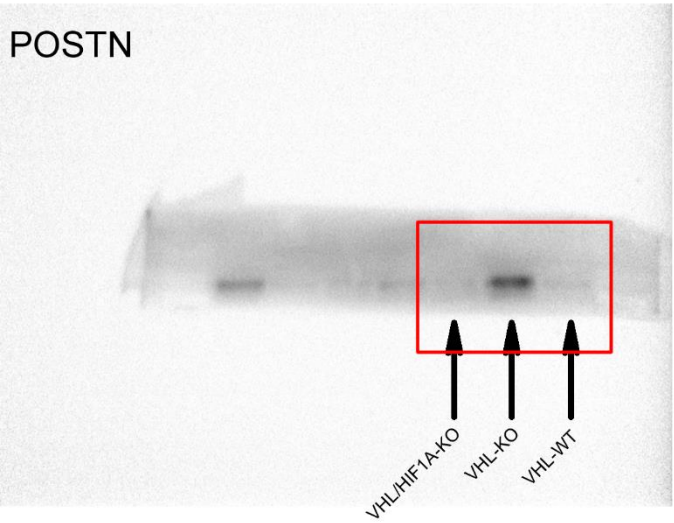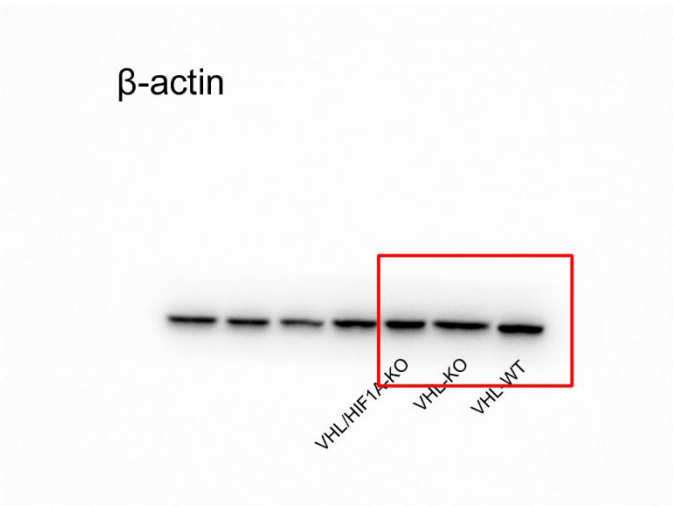

Fig. 6a

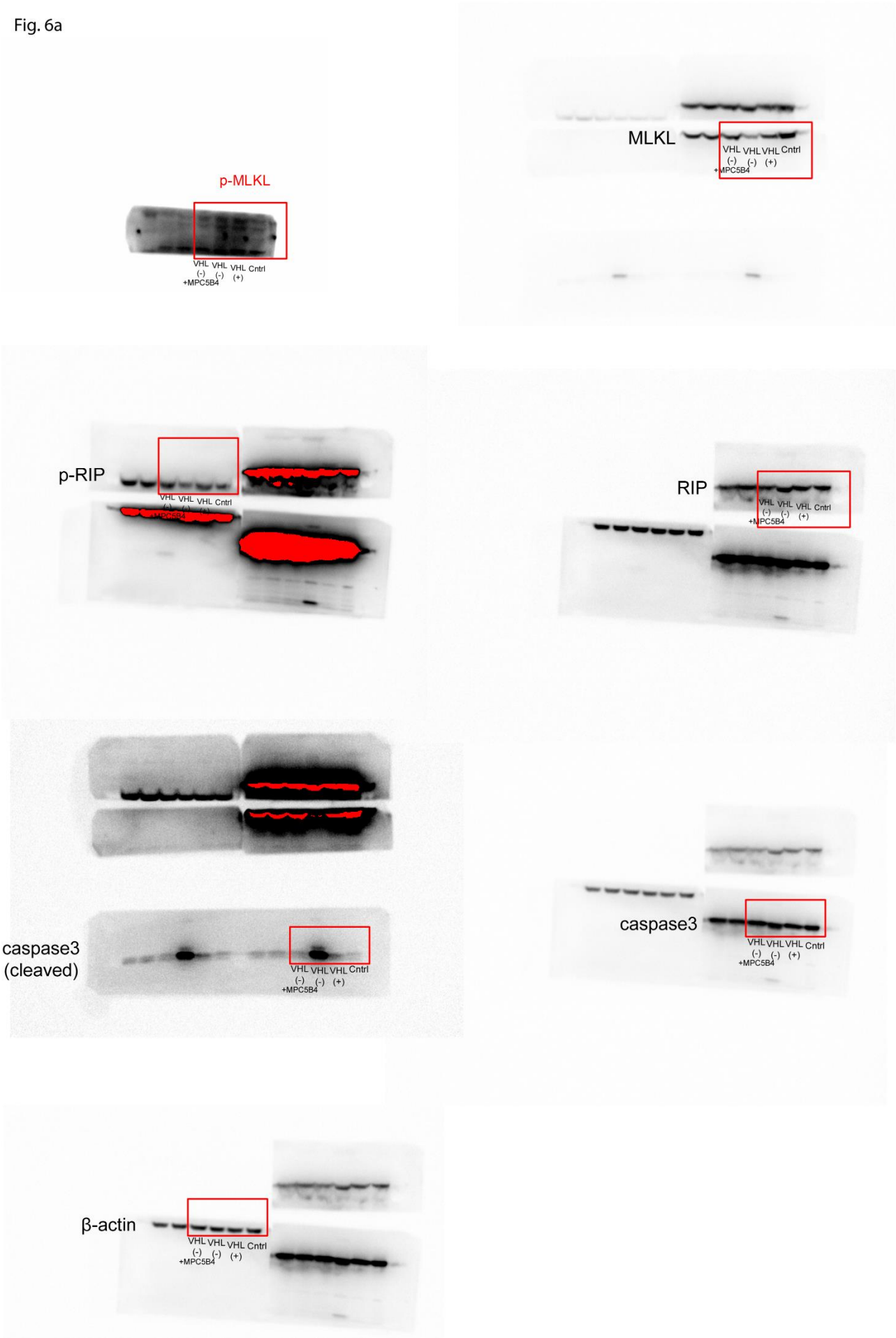

Fig. S1f

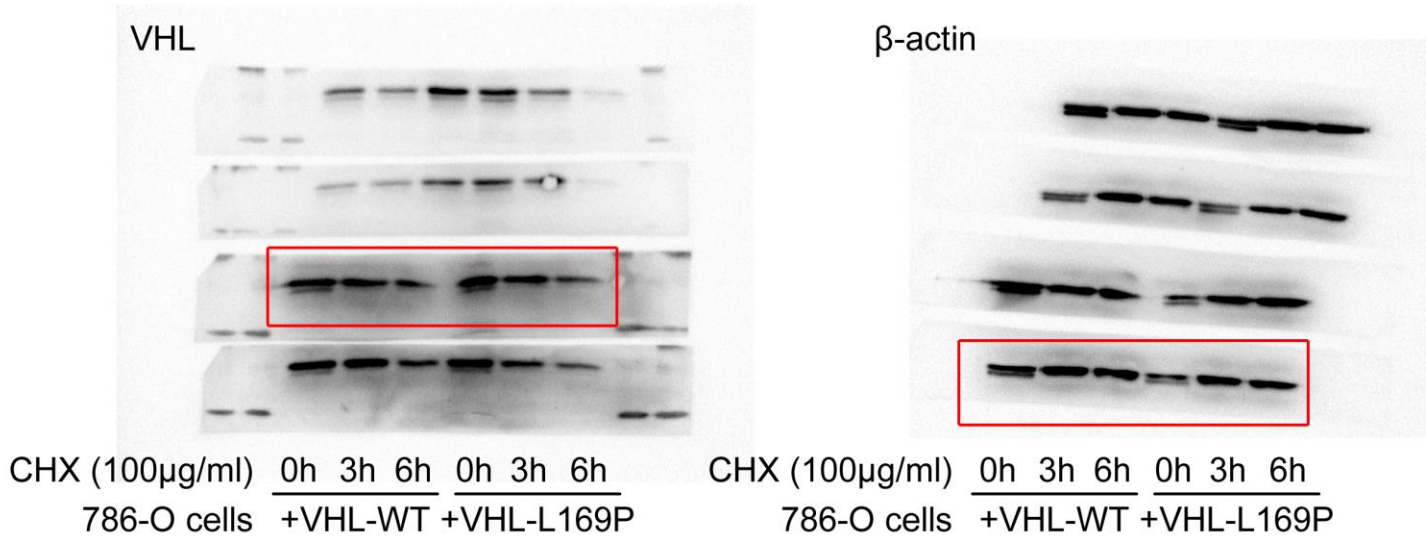

Fig. S1g

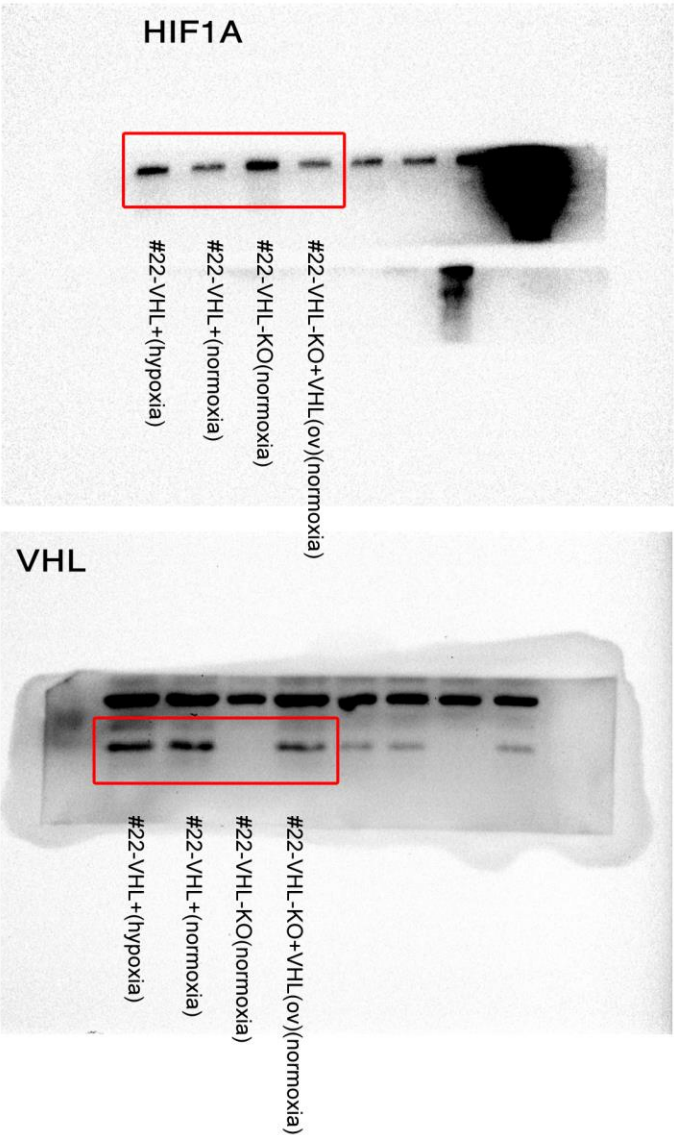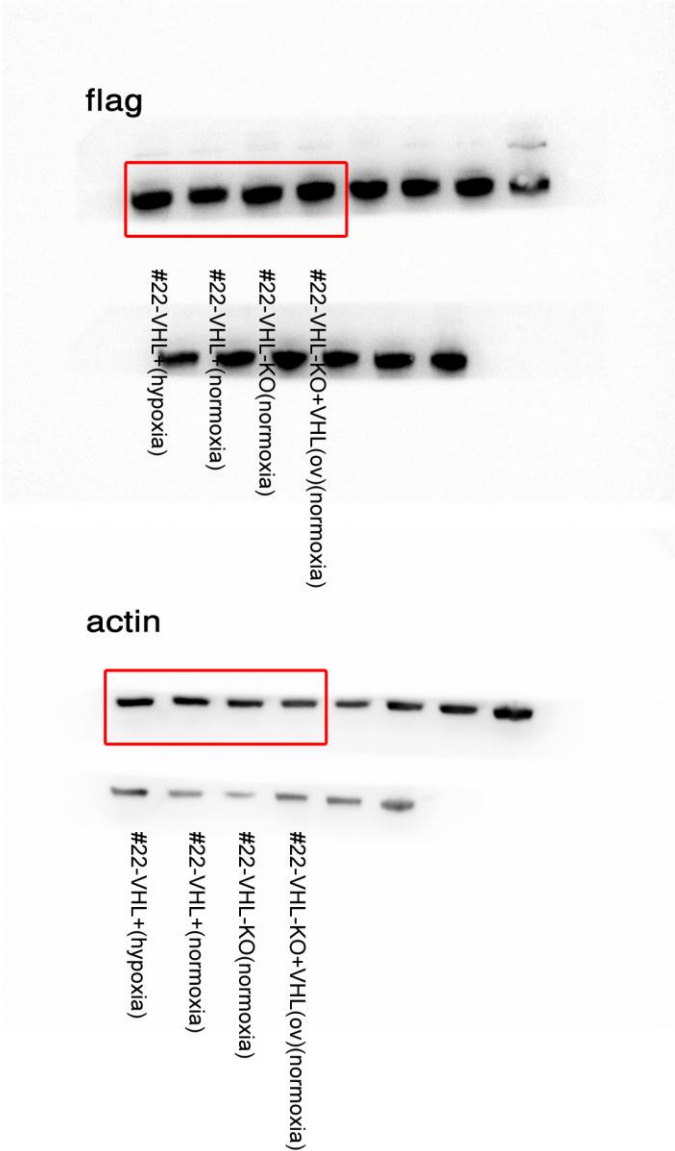

Fig. S1h

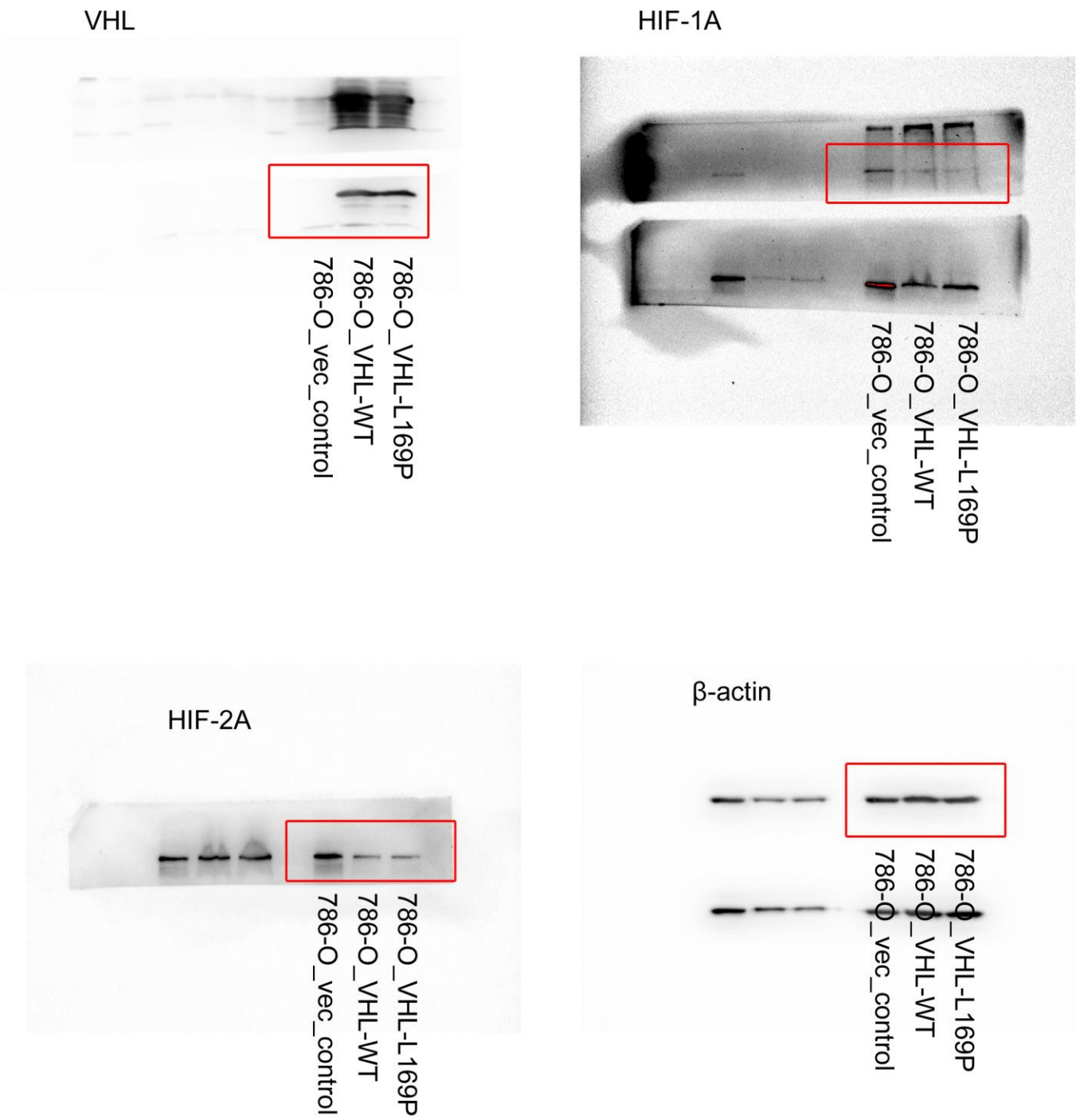

Fig. S1i

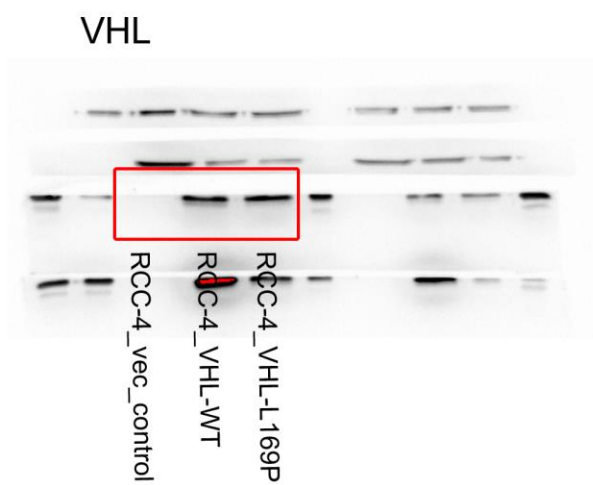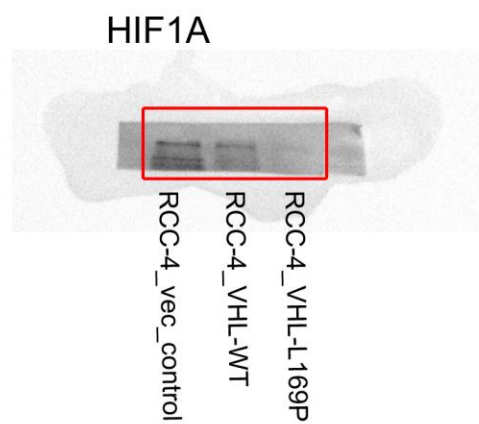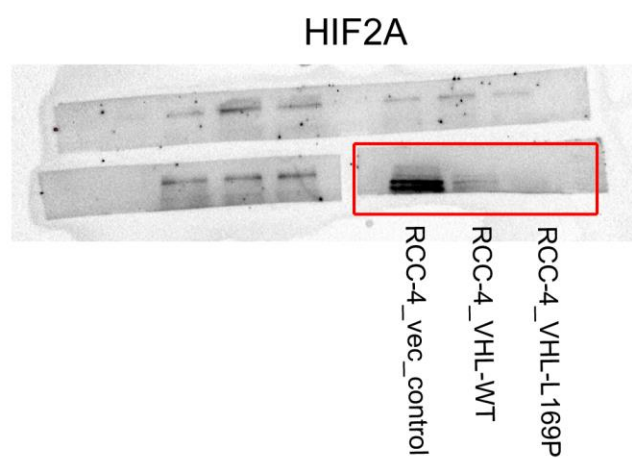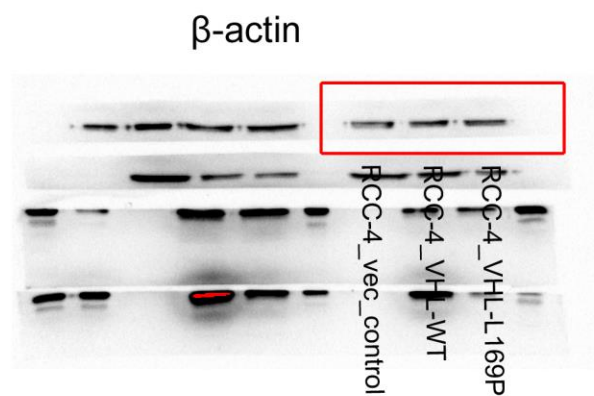

Fig. S4j

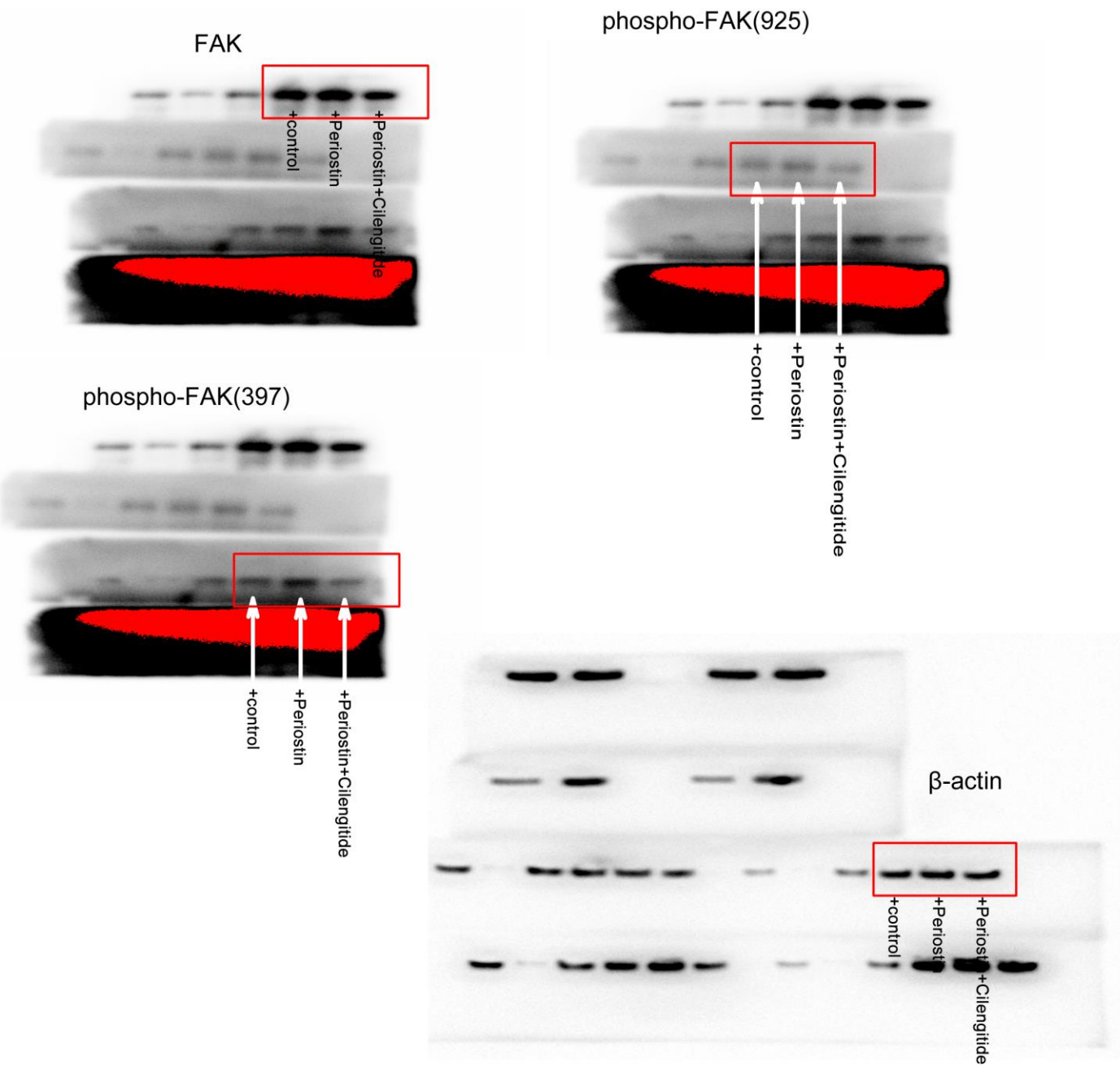

Supplement: Supplementary file 2 — Original uncropped WB films [file 41392_2023_1362_MOESM2_ESM.pdf]
